# Microenvironmental reorganization in brain tumors following radiotherapy and recurrence revealed by hyperplexed immunofluorescence imaging

**DOI:** 10.1038/s41467-024-47185-9

**Published:** 2024-04-15

**Authors:** Spencer S. Watson, Benoit Duc, Ziqi Kang, Axel de Tonnac, Nils Eling, Laure Font, Tristan Whitmarsh, Matteo Massara, iMAXT Consortium, Johanna A. Joyce, Spencer S. Watson, Tristan Whitmarsh, Bernd Bodenmiller, Bernd Bodenmiller, Jean Hausser, Johanna A. Joyce

**Affiliations:** 1 Department of Oncology, University of Lausanne, Lausanne, Switzerland; 2 Ludwig Institute for Cancer Research, University of Lausanne, Lausanne, Switzerland; 3 Agora Cancer Research Center, Lausanne, 1011 Switzerland; 4 L. Lundin and Family Brain Tumor Research Center, Departments of Oncology and Clinical Neurosciences, Centre Hospitalier Universitaire Vaudois, Lausanne, 1011 Switzerland; 5 Department of Cellular and Molecular Biology, Karolinska Institutet and SciLifeLab, Stockholm, Sweden; 6 Department of Quantitative Biomedicine, University of Zurich, Zurich, Switzerland; 7 Institute for Molecular Health Sciences, ETH Zurich, Zurich, Switzerland; 8 École Polytechnique Fédérale Lausanne, Lausanne, Switzerland; 9 Machine Intelligence Laboratory, Department of Engineering, University of Cambridge, Cambridge, UK; 10 Cancer Research UK, Cancer Grand Challenges iMAXT Consortium, University of Cambridge, Cambridge, UK

**Keywords:** Cancer microenvironment, Fluorescence imaging, Image processing

## Abstract

The tumor microenvironment plays a crucial role in determining response to treatment. This involves a series of interconnected changes in the cellular landscape, spatial organization, and extracellular matrix composition. However, assessing these alterations simultaneously is challenging from a spatial perspective, due to the limitations of current high-dimensional imaging techniques and the extent of intratumoral heterogeneity over large lesion areas. In this study, we introduce a spatial proteomic workflow termed Hyperplexed Immunofluorescence Imaging (HIFI) that overcomes these limitations. HIFI allows for the simultaneous analysis of > 45 markers in fragile tissue sections at high magnification, using a cost-effective high-throughput workflow. We integrate HIFI with machine learning feature detection, graph-based network analysis, and cluster-based neighborhood analysis to analyze the microenvironment response to radiation therapy in a preclinical model of glioblastoma, and compare this response to a mouse model of breast-to-brain metastasis. Here we show that glioblastomas undergo extensive spatial reorganization of immune cell populations and structural architecture in response to treatment, while brain metastases show no comparable reorganization. Our integrated spatial analyses reveal highly divergent responses to radiation therapy between brain tumor models, despite equivalent radiotherapy benefit.

## Abstract (web summary)

Improved imaging techniques are required to help advance our understanding of the complex role of the tumour microenvironment (TME). Here, the authors develop a high-throughput, highly multiplexed tissue visualisation workflow and demonstrate its utility by characterising the response of the TME to radiotherapy in preclinical models of glioblastoma.

## Introduction

Glioblastoma is the most common and most aggressive primary brain tumor in adults, with an incidence rate of up to 5 per 100,000 people^1^. Standard-of-care treatment, consisting of surgical resection, ionizing radiation (IR), and temozolomide-based chemotherapy, leads to only a transient response. Consequently, median survival is just over 14 months, and the five-year survival is <5%^2,3^. This poor prognosis is due to tumor recurrence in nearly all cases, emphasizing the need for a deeper understanding of the mechanisms driving therapeutic resistance.

Previous studies have investigated how glioblastoma responds and recurs following both IR therapy and macrophage-targeted immunotherapy^4–10^. One key mediator of resistance to treatment is tumor-cell heterogeneity and plasticity, resulting in IR-resistant subpopulations that can rapidly repopulate the lesion^11–13^. However, another critical aspect is how the tumor microenvironment (TME) responds to IR, as this can modulate the efficacy of IR through several mechanisms^14–16^. We previously revealed how different treatment modalities induce substantial changes in immune cell populations in murine models of glioblastoma^4–7^, which are related to eventual tumor recurrence. However, due to the substantial number of cellular and extracellular interactions in the TME, and consequently the highly focused nature of previous studies, many of the general mechanisms driving these pro-survival roles remain uncertain. It is thus increasingly evident that there is a need to investigate the TME as a whole, to interrogate the interplay of all the major cell types with each other and with structural features in the tumor ecosystem.

One challenge in studying the entire TME in all its complexity is the substantial amount of different cell types, and the high number of markers needed to delineate and identify each cell type in a single sample. The typical methods used to explore cellular heterogeneity in the TME include multiparameter flow cytometry and single-cell RNAseq. However, both methods disassociate the tissue, thereby losing all spatial context of cellular interactions, in situ cellular morphology, and correlation with tissue architecture and ECM. For this reason, high-dimensional imaging techniques have become increasingly vital to the study of the TME. Imaging mass cytometry (IMC)^17^, tissue cyclic immunofluorescence (t-CycIF)^18^, Multiplexed Ion Beam Imaging by Time of Flight (MIBI-TOF)^19^, MALDI mass spectrometry imaging (MALDI-IMS)^20^, and oligo-conjugated antibody cyclic immunofluorescence (Ab-oligo cyCIF)^21^ are among the current approaches^22,23^ for highly multiplexed tissue imaging capable of assessing 20–100 markers on a single tissue section.

While these techniques represent powerful and well-validated methods of examining the spatial topography of the TME, they are not universally suited to all applications. IMC has relatively small imaging areas of 1 mm^2^ which have a spatial resolution of approximately 1 µm (equivalent to ~10X magnification). MIBI-TOF can achieve much higher resolutions, up to 200 nm, but this comes with a significant trade-off in lengthy image acquisition times. MALDI-IMS can easily image whole-slide sections, but with a maximum resolution of only 10 µm^24^. This limits the ability to study tumors with structural features that exceed these dimensions, or with cellular interactions involving small dendritic extensions, for example. In addition, commercial mass cytometry imaging and Ab-oligo cyCIF solutions have a substantial initial adoption cost, and require custom-conjugated antibodies, both of which can be prohibitively expensive, or challenging to conjugate if the required markers are not commercially available. A significant additional limitation of these approaches is the low throughput, with typical automated workflows allowing imaging of <10 slides per week. Immunofluorescence (IF)-based multiplexed approaches use off-the-shelf commercially available antibodies, but have many of their own challenges. Current highly multiplexed imaging approaches require repeated rounds of staining, imaging, and stringent stripping or quenching of markers which can damage tissue samples and the protein epitopes targeted by antibodies. Moreover, while several of these antibody removal methods are well validated in formalin-fixed paraffin-embedded (FFPE) tissue, they can damage or destroy lightly fixed frozen tissue.

In this study, we present a complete workflow for whole-slide highly multiplexed tissue imaging, which we term Hyperplexed Immunofluorescence Imaging (HIFI), to address multiple limitations of existing methodologies. By requiring no bespoke reagents or equipment, using commonly available reagents, and utilizing open-source analysis approaches we aim to address the issues of cost and accessibility of high-dimensional imaging for a broad scientific community. We apply this workflow to the study of TME response to focalized IR in a genetic mouse model of glioblastoma. We employ a 45-marker panel to analyze a sample set of tissues collected prior to treatment, 7 days post-IR, or at the point of tumor recurrence to study the spatial microenvironment response to treatment. To investigate the extent of microenvironmental response depending on the tumor type, we also compare the glioblastoma model to a breast-to-brain metastasis model at comparable volumes and timepoints. This comparison reveals substantial TME reorganization in glioblastomas in response to IR treatment, and the consistent generation of survival-promoting spatial niches at 7 days post-IR. Conversely, brain metastases do not show spatial reorganization or significant debulking in response to IR, despite both tumor types receiving equivalent survival benefit from the therapy.

## Results

### Overview of HIFI spatial analysis workflow

**Fig. 1:**
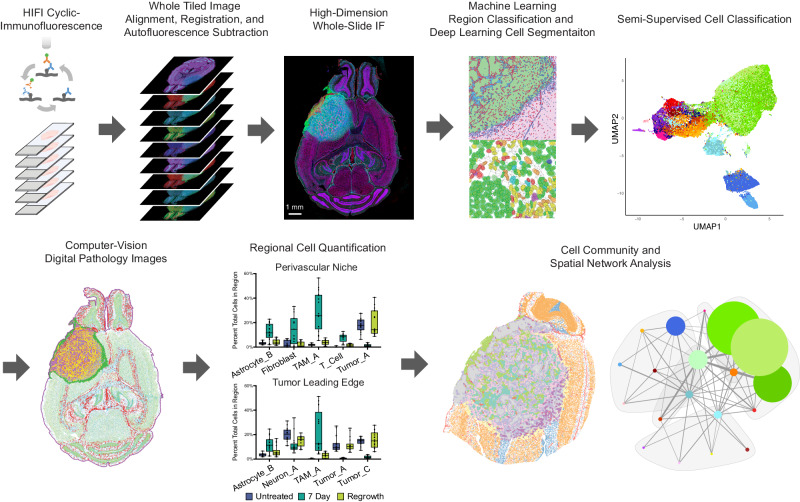
Overview of Hyperplexed Immunofluorescence Imaging (HIFI) Workflow. Experimental workflow of cyclic immunofluorescence staining, followed by image processing, alignment, and registration to create 45+ dimensional images across whole-slide sections. Specific domains within HIFI images of tumors were automatically annotated using trained machine-learning classifiers, and individual cells were segmented with deep-learning object detection. Single-cell objects were annotated as individual cell types with semi-supervised classification and mapped back onto images to create highly-annotated digital pathology images. Images were analyzed for regional cellular composition and spatial organizational analysis. The HIFI workflow is scalable to over 100 simultaneous sections for high-throughput spatial experiments.

We purposely designed HIFI to be straightforward to implement with open-source software and without the need for specialized lab equipment. For these reasons, all IF staining was performed manually using standard benchtop methodology and reagents, and all imaging was performed with conventional commercial slide-scanning microscopes. Figure 1 provides a graphical overview of the typical HIFI workflow, showing the process of cyclic immunofluorescence imaging, whole-slide image alignment and registration, machine-learning structural annotation, deep-learning cell segmentation, and clustering-based cell classification to generate highly annotated digital pathology images for spatial analysis.

The workflow time course for a single experiment is dictated by the size of the antibody panel, how many markers can be multiplexed in each imaging round, and the speed of image acquisition. Multiple days are required to complete a full hyperplexed panel. However, as most modern slide-scanning fluorescence microscopes have capacities for 80–100 slides, the ability to scale up to high-throughput offsets the total time required for an experiment when compared to other highly multiplexed approaches. 40× and 60× magnifications across tissue sections are also feasible, however, this would come with a tradeoff of increased scanning time.

A core component of the HIFI variant of cyclic-IF that we present here is the gentle antibody removal, coupled with the avoidance of crosslinking between fluorophores and tissue in the imaging stage. The most disruptive step in other cyclic-IF approaches is typically the elution of marker signal between imaging rounds. These methods use either stringent acidic buffers to strip antibodies^25^, or a combination of light and hydrogen peroxide to inactivate fluorophores^18^, both of which can potentially damage lightly-fixed and fragile tissue samples. HIFI utilizes a thiol-based elution buffer to reduce the disulfide bonds of bound antibodies, releasing the antibodies from the tissue without damaging tissue integrity. Thiol-based elution permits efficient yet gentle removal of standard off-the-shelf primary and secondary antibodies. Elution was further optimized for tissue sections by using an appropriate pH and a brief 3-min elution step. This method was previously employed in cell culture using the iterative indirect immunofluorescence imaging (4i) approach^26^, and was adapted herein for use in tissue sections for the first time. The basis of 4i is the observation that high-energy light from fluorescence microscopy light sources can result in indirect oxidative crosslinking of fluorophores to the tissue, preventing efficient elution. Inhibition of this crosslinking through oxygen radical scavengers, or reduced light energy, can thereby improve antibody elution at less stringent conditions. To address this, we purposely optimized HIFI for low-power LED fluorescent light sources, to prevent crosslinking while maintaining robust fluorescence signals. These optimizations additionally allow for the use of standard glass coverslips and glycerol-based mounting media while imaging, rather than specialized mounting solutions. A further benefit of low-power imaging is reduced fluorescence spillover between channels when combined with appropriate bandpass filters.

The workflow of immunostaining, imaging, coverslip removal, and elution are repeated until all rounds of markers are imaged. Following this non-destructive imaging, samples can be stained with hematoxylin and eosin (H&E) for future analysis and stored long-term, or used for additional experiments, such as proteomics. The post-processing, alignment, and analysis of HIFI-generated images are discussed in the following use cases.

### Generation of IR treated brain tumor samples and antibody labeling panel

Glioblastomas present with a high degree of heterogeneity, with spatial features that can cover substantial distances. Because of this, selecting individual regions of interest (ROIs) for high-dimensional image analysis can impart a substantial potential for selection bias. For this reason, to robustly investigate how the cellular and structural topography of glioblastoma responds to IR therapy, we endeavored to develop a high-dimensional spatial analysis pipeline for whole tissue sections that was not limited by sample size or preparation, and which could achieve subcellular resolution and semi-quantitative protein detection.

We utilized the RCAS-hPDGF-B; Nestin-Tv-a; Ink4a/Arf KO genetically engineered mouse model (GEMM) to model glioblastoma^27,28^. This model drives neoplastic transformation in nestin-expressing neural progenitor cells via overexpression of platelet-derived growth factor-B (Pdgfb), resulting in glioblastomas that mimic the human proneural phenotype, and with a fully intact immune system^27,28^. In addition, the transformation includes expression of green fluorescent protein (GFP) in Pdgfb-overexpressing cells, to fluorescently label and track the resulting tumor cells. These gliomas are termed PDGfp herein. For a comparative dataset we selected an orthotopic immune-competent model of breast-to-brain metastasis (BrM) utilizing a luminal HER2 + MMTV-PyMT-derived cell line^5,29^ injected at matched cranial coordinates as used for initiation of the PDGfp model.

**Fig. 2:**
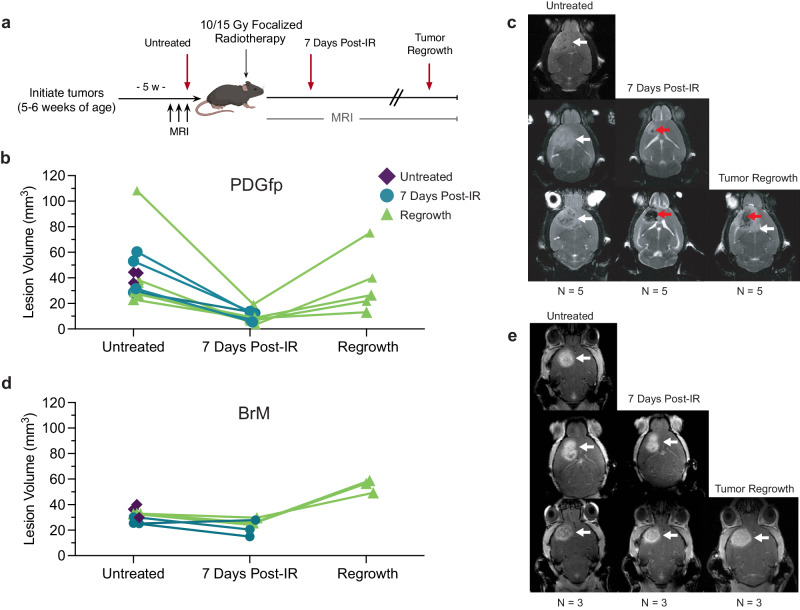
Irradiation Treatment Sample Collection and Antibody Panel. **a** PDGfp and BrM tumors were initiated in NTVA-Ink4a/Arf−/− and C57Bl6 mice respectively, and monitored by MRI. Experimental endpoints (red arrows) are indicated for each group. Biweekly MRI monitoring tracked the process of regression and recurrence for each mouse following IR for **b**, **c** PDGfp (*n* = 5 mice for each treatment group) and **d**, **e** BrM tumors (*n* = 3 mice for each treatment group. Upon tumor detection, mice were grouped into cohorts and collected as either (i) ‘untreated’ samples, (ii) treated with 10 Gy (PDGfp) or 15 Gy (BrM) focalized irradiation (IR) therapy and harvested 7 days post-IR, or (iii) treated with 10 or 15 Gy IR and harvested upon tumor regrowth. White arrows indicate the tumor in both tumor types, red arrows indicate post-IR lesion in PDGfp tumors. Source data are provided as a Source data file.

PDGfp or BrM tumors were initiated in mice at 5-6 weeks of age, and animals monitored weekly by magnetic resonance imaging (MRI) to screen for tumor formation and growth (Fig. 2a–c). PDGfp tumors were initiated in both male and female mice, while breast-BrM were injected only in female mice. Once PDGfp tumors reached a volume of >20 mm^3^, they were either harvested (*n* = 5), or treated with a single focalized 10 Gy IR dose (*n* = 10). BrM were similarly monitored until tumors reached comparable volumes, and then either harvested (*n* = 3), or treated with a single focalized 15 Gy IR dose (*n* = 6) (Fig. 2d). Appropriate radiation doses for both tumor types were based on previous data that assessed efficacious IR doses in these models (ref. ^5^, and Wischnewski, [..], Joyce, manuscript in preparation). Critically, despite the lesser tumor volume reduction in BrM, both models ultimately showed equivalent survival benefit from these focalized IR doses. Comparisons between treated samples in this study and historical data for untreated subjects were completely in keeping with previous survival data for these models^4,5,29^ (Wischnewski, [..], Joyce, manuscript in preparation). At 7 days post-IR, MRI was performed on all mice, and tumor-bearing brains were harvested for *n* = 5 PDGfp and *n* = 3 BrM mice. MRI monitoring was continued bi-weekly for the remaining mice until tumor recurrences were detected. Brains were subsequently harvested (PDGfp *n* = 5, BrM *n* = 3) and tissues embedded for cryo-sectioning. Volume measurements showed substantial reductions in tumor size in PDGfp tumors 7 days post-IR treatment, and a subsequent increase in volume at the point of tumor regrowth (Fig. 2b, c). BrM tumors showed more modest reductions in tumor volumes in response to IR, and a similar increase in volume at the point of regrowth (Fig. 2d).

Following cryo-sectioning of brain tissue samples, at least three replicate sections spaced at regular intervals across the entire Z-depth of each sample were used to account for intratumoral heterogeneity. In total, 87 whole-brain tissue sections were prepared for the imaging pipeline. 45 markers were chosen to interrogate the PDGfp tumor samples at each treatment stage. The antibody panel was based on previous studies characterizing the TME of murine glioma models by flow cytometry and other methods^4–6,30^. Various markers were selected to label each of the predominant tumor, glial, vascular, and immune cell types (Table 1). In addition, multiple antibodies were selected to label ECM structures relating to the healthy brain, perivascular niche, and treatment-induced fibrosis. An additional series of markers was used for the purpose of training machine learning models and for quality control. A subset of 29 of these markers was selected to interrogate BrM samples, to which 4 new antibodies were added to specifically analyze breast cancer heterogeneity (Supplementary Table 1).

**Table 1:** PDGfp HIFI marker panel

Tumor	
GFP	Pan tumor
Olig2	OPC
Sox9	Glial precursor
Sox2	Glial precursor

Immune	
CD3	Pan T cell
CD8a	Cytotoxic T cell
Iba1	Macrophage
CD68	Macrophage
P2YR12	Microglia
CD45	Pan leukocyte
CD206	Meningeal
Ly6b	Neutrophil
S100A8	Neutrophil activation
MPO	Neutrophil activation

ECM	
Tenascin-C	Fibrotic ECM
Laminin	Vascular ECM
Periostin	Non-structural ECM
CSPG5	Parenchymal ECM
Fibronectin	Fibrotic ECM
Collagen I	Fibrotic ECM
Collagen IV	Fibrotic ECM

Neurons	
FoxP1	Neural stem cell
NeuN	Pan neuronal
NF-H	Neurofilament

Vascular	
ER-TR7	Reticular fibroblast
PDGFRB	Pan perivascular cells
CD31	Endothelial
αSMA	Mural cells
CD13	Pericyte
VE-Cad	Endothelial

Glial	
Vimentin	Astrocyte subset
GFAP	Astrocyte activation
Podoplanin	Reactive gliosis
S100B	Pan astrocyte
Desmin	Astrocyte subset
NG2	OPC

Cell State	
Ki67	Proliferation
CC3	Apoptosis
HIF1a	Hypoxia
Lamin AC	Nuclear envelope

Functional	
RedDot2	QC
WGA	Machine learning
αTubulin	Machine learning
Phalloidin	Machine learning

List of multiplexed markers used specifically for PDGfp glioblastoma samples, indicating category of marker, marker name, and marker target.

A critical component in designing the HIFI panel was to determine which antibodies could be multiplexed together, as well as the ability to reliably label their target epitope following multiple rounds of staining, imaging and elution. Antibody validation was performed using existing methodology (Supplementary Note 1)^18,26^, which entails comparisons of marker specificity in previously cyclic-stained tissue versus unstained sections. To verify that markers are removed between imaging rounds, control experiments were performed to repeatedly label and image multiple tissues using a set of primary and secondary antibodies. Markers were eluted following imaging, and tissues were re-stained with only secondary antibodies and reimaged. This process was repeated multiple times to determine single-cell mean fluorescence intensity (MFI) for markers at each stage (Supplementary Fig. 1). These data also show which marker intensities are maintained over multiple elution rounds, which decrease, and which even increase over rounds, thereby informing the sequential order of markers in the panel. Markers were thus arranged into 12 individual multiplexed panels based on their labeling efficiency and species cross-reactivity (Table 1, Supplementary Fig. 1a).

### Whole-slide image alignment and registration

Raw tiled image data from all rounds were stitched together for each image with affine transformation (Zeiss Zen software package) to create seamless whole-section images. Background fluorescent signal was removed using the rolling-ball algorithm.

**Fig. 3:**
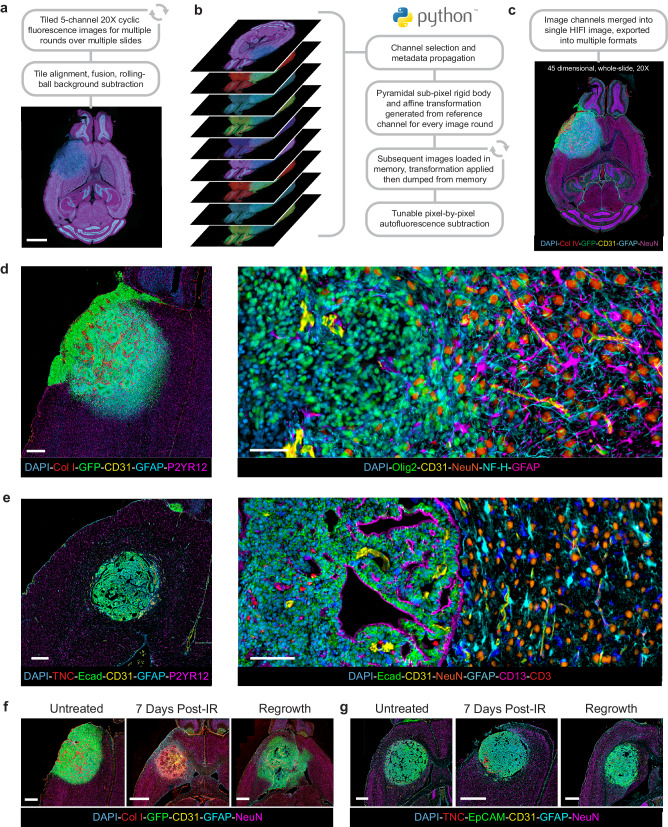
HIFI Alignment. **a** Image processing of raw image data consisted of tile stitching and flat-field correction, followed by rolling-ball background subtraction. Scale bar = 2 mm. **b** Panel depicts the repeated steps of the “HIFI Alignment” tool to align cyclic IF images while avoiding memory limitations and removing endogenous tissue autofluorescence. **c** Aligned and registered images were then merged into a single 45+ dimension whole-slide image. Images for **d** PDGfp and **e** BrM were cropped to encompass the entire tumor region and surrounding brain. Scale bars = 500 µm. Representative images of (**f**) PDGfp and (**g**) BrM samples from untreated, 7 days post-IR, and regrowth tumors. Scale bars = 400 µm.

In cyclic fluorescence slide scanning, each round of imaging may not necessarily be in the same exact position or orientation due to stage drift and slide placement, so multiplexed images cannot be simply merged directly. Whole-section images on this scale could not be aligned with publicly or commercially available tools due to memory limitations and array-size limits. Therefore, we had to develop an automated software tool in Python to align and register all rounds of multiplexed imaging into a single hyperplexed image. The “HIFI Alignment” tool utilizes existing algorithms for pyramidal sub-pixel image alignment based on pixel intensity^31^, and implements them in Python using an image handling strategy to bypass memory limitations due to image array-size, bit-depth, or the number of channels (Fig. 3a).

This strategy enables whole-slide rigid-body or affine alignment of 45+ channel images, which is only limited by the physical RAM available on a workstation (Fig. 3b, c). Whole mouse brain sections were aligned using consumer-grade workstations with 256 GB of RAM, while image subsets of whole PDGfp tumors could be aligned on standard computers with 64 GB of RAM. HIFI Alignment includes a CZI reader and writer for Axio Scan.Z1 images, but can also accommodate images in multiple TIFF formats.

An important feature of the HIFI Alignment tool is the ability to perform pixel-by-pixel removal of tissue autofluorescence from all channels to prevent any contamination of marker signals in subsequent rounds of imaging. Imaging of tissue samples in all channels prior to antibody labeling, as was performed with the PDGfp and BrM IR-treated samples, creates an image of overall tissue autofluorescence which is then subtracted from respective channels in each round of imaging. Autofluorescence subtraction was then performed for all BrM samples, but not for PDGfp samples - so as to not remove the endogenous GFP signal in the tumor cells.

The HIFI methodology was specifically developed to work with challenging sample types that perform poorly with other highly multiplexed imaging techniques. To validate that HIFI also works in more standard sample types, we performed additional HIFI experiments across multiple murine organ FFPE samples (Supplementary Fig. 2).

Following imaging, sections were cropped to the area of interest. Whole-slide imaging facilitated selection of regions for analysis that encompassed the entire tumor area and large regions of the surrounding brain, thereby eliminating sample bias within the tumor. This workflow generated sixty PDGfp images in 45-dimensions, and twenty-seven BrM images in 33-dimensions (Fig. 3d, e). Untreated and 7-days post-IR PDGfp tumors were found to be consistent across replicates in terms of position and morphology, while regrowth gliomas showed extensive morphological heterogeneity (Fig. 3f, Supplementary Fig. 3a). Conversely, BrM tumors were observed to be highly consistent across all replicates and treatment conditions (Fig. 3g, Supplementary Fig. 3b).

### Machine learning structural annotation and deep learning cell segmentation

**Fig. 4:**
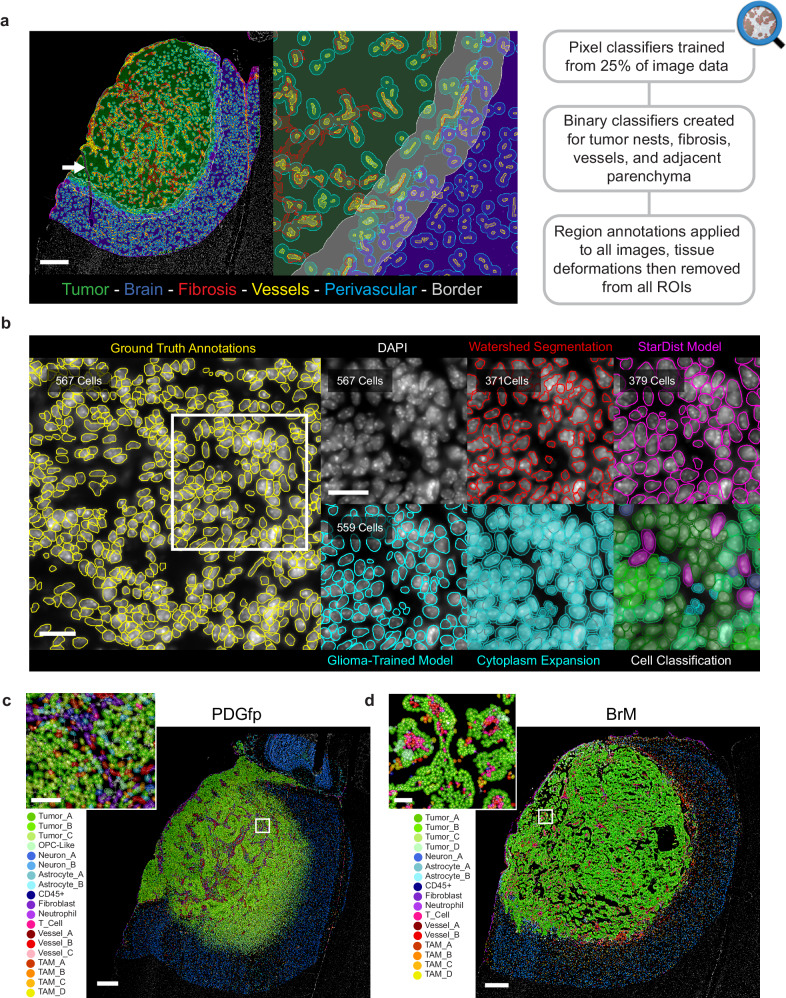
Region Annotation, Cell Segmentation and Classification. **a** Binary pixel classifiers were trained for multiple spatial regions (as indicated) in QuPath on a subset of image data. Tissue deformations and imaging errors were removed from regions of interest (ROIs) during quality control checks (white arrow indicates a representative example of a tissue tear). Scale bar = 500 µm. **b** Nuclei in murine glioblastoma samples were manually annotated to train StarDist nuclear detection models. Detection accuracy was compared to threshold-based watershed segmentation and the publicly available StarDist model for immunofluorescence (DSB_Heavy). Segmented nuclei were expanded by 2.5 µm to capture cell cytoplasm and classified by semi-supervised cell classification. Scale bars = 50 µm. Region annotation, cell segmentation, and cell classification were applied independently to all (**c**) PDGfp and (**d**) BrM HIFI images to generate fully annotated digital pathology images. Scale bars = 400 µm.

HIFI images were first reviewed for all channels to identify any imaging aberrations, such as dust contamination or tissue deformations from sectioning, for the purpose of excluding these areas from downstream analysis (Fig. 4a). During this critical quality control review, HIFI images that did not meet stringent criteria were excluded from the study, resulting in *n* = 81 images for subsequent in-depth analysis. Images were analyzed with the open-source digital pathology suite QuPath^32^. The ability to image an entire tumor area with subcellular resolution facilitated the correlation of individual cell types with larger features of tumor architecture. To analyze this topographical heterogeneity, we trained an AI pixel-classifier model with pathological annotations based on multiple cellular and ECM features, and then applied each model for each feature across all images (Fig. 4a). Tissue regions within each sample were automatically annotated to identify lesion boundaries, tumor nests, vasculature, areas of fibrosis, and modified to delineate perivascular niches, the surrounding brain, and tumor-brain interface border.

We then performed cell segmentation for single-cell measurements of MFI, cell size, and localization. However, glioma tissue contains densely packed irregular nuclei, and DAPI staining of nucleic acids can additionally create non-uniform labeling across nuclei (Fig. 4b). This combination of factors dramatically reduced the accuracy of intensity-based watershed algorithms for nuclei segmentation in these tissues. (Fig. 4b). For this reason, we instead employed the supervised deep-learning algorithm StarDist^33^ implemented in QuPath. StarDist utilizes U-Net^34^, a convolutional neural network (CNN) designed for biomedical image analysis. As such, StarDist requires annotated ‘ground truth’ images to train the CNN to learn to accurately predict object probabilities. Ground truth nuclear annotations were manually performed on 144 large images of multiple murine tissues stained with DAPI. Our murine training dataset included multiple samples from PDGfp tissues, breast-BrM, lung-BrM, breast tumors, human breast tumor xenografts, and healthy tissue from brain, mammary glands, and lungs. Additional images were generated via image transformations using transposition, flipping of the axes, as well as random changes of signal intensity, to encourage the CNN to be robust to these transformations. Transfer learning from the published StarDist immunofluorescence model^33^ was also performed to further increase model accuracy. We used this approach to generate two robust deep-learning nuclei segmentation models; one specifically trained on only PDGfp tumors for enhanced accuracy, and another trained on the entire data set for broad applicability to any mouse tissue (Fig. 4b). The publicly released DSB_Heavy StarDist cell segmentation model was found to have an accuracy of 68.86% on our training data, while our glioma-specific model had an accuracy of 74.96% (Fig. 4b). Accuracy was further enhanced in the dataset by filtering all cells with a detection probability score of <0.5.

Segmentation was performed with StarDist using our murine-trained model to identify each nucleus as a vector object, and each object was given an additional maximum expansion of 2.5 μm to measure cytoplasmic marker signals (Fig. 4b). Cell size, shape, MFI, and XY location were measured for every cell. Further spatial measurements were performed following single-cell MFI and morphometric measurements. Additionally, the distance of each cell to the nearest region annotation border was recorded to measure cell proximity to all structural features within the image.

### Clustering based semi-supervised cell classification

A potential drawback of using cyclic immunofluorescence for high-dimensional imaging is the low dynamic range, or depth, of marker signals compared to mass spectrometry-based approaches. This can create challenges for standard clustering-based approaches of unsupervised cell classification, especially when some markers have low intensity relative to other markers. A further challenge to employing computationally intensive clustering algorithms is that applying them to very large single-cell HIFI datasets (such as the PDGfp dataset containing ~7 × 10^6^ cells) is the time required for analysis, which can be >1 week for a single dataset. Therefore, to achieve distinct clusters for cell classification despite low signal dynamic range, we next optimized a computationally efficient FlowSOM approach^35^.

MFIs for markers unique to specific cell types were measured for the whole-cell area or the nuclear area, depending on the cell biology of each marker (Supplementary 2). This enhanced cluster specificity and reduced signal spillover to neighboring cells. Each MFI used for classification was scaled from 0 to 1, and clipped to the 99.7 percentile. Cells were filtered based on size to remove fragments, and each PDGfp and BrM dataset were clustered independently across all treatment types with FlowSOM to maintain uniformity. FlowSOM was set to produce 100 unique nodes for each dataset. Hierarchical clustering of marker MFIs was used to cluster nodes into cell type annotations (Supplementary Fig. 4a–c).

Cellular annotation of unbiased clusters based on previous biological knowledge of the TME resulted in semi-supervised cell classification (Fig. 4c, d, Supplementary Fig. 5a, b). To maintain aspects of cellular heterogeneity we used generic nomenclature, such as Tumor_A or Tumor_B, to provide general classifications while still maintaining the unbiased heterogeneity that was revealed by cluster analysis. In particular, tumor cells, TAMs, astrocytes, and vasculature presented with consistent heterogeneity in both the PDGfp and BrM datasets. Four tumor cell clusters were identified in PDGfp samples, Tumor_A-C and the category OPC-Like which could not reliably be distinguished from normal oligodendrocyte precursor cells (OPCs) due to their known phenotypic similarity. These subtypes were stratified based on their expression of GFP, Olig2, Sox2, and Sox9 (Supplementary Fig. 5c). We further analyzed the proportion of Ki67 positivity, showing that tumor clusters A, B, and C had equivalent proportions of proliferating cells in untreated and regrowth tumors (Supplementary Fig. 5d). OPC-Like cells showed the least proliferation, but still sufficient levels to indicate that this population included neoplastic cells, despite their lack of the GFP tumor cell marker (Supplementary Fig. 6a). This is consistent with the reported ability of glioblastomas to recruit non-transformed cells into the TME^36^. Interestingly, the Tumor_A phenotype, which had the highest expression of GFP, was the most impacted by IR treatment in terms of proliferation, showing almost no proliferation at 7-days post treatment (Supplementary Fig. 5c, d). This phenotype is consistent with quiescent residual glioma cells that are both radio- and chemo-resistant^37^.

In BrM, we also identified four tumor cell clusters, termed Tumor_A-D, stratified based on expression of EpCAM, cytokeratin 8, cytokeratin 14, and E-cadherin (Supplementary Fig. 5e). Each BrM tumor cluster showed almost complete loss of proliferation following IR (Supplementary Fig. 5f). The percent of proliferating cells in regrowth BrM did not match that of untreated tumors, but this likely does not reflect long-term alterations in proliferative capacity caused by treatment. For animal welfare reasons, and to maintain comparable tumor sizes, regrowth BrM were harvested when progression was measurable by MRI, not at the point that their growth curve matched that of untreated tumors. Tumor clusters in BrM showed consistent spatial localization across treatment types, with the type A phenotype comprising the majority of the tumor core, while type B was dominant in areas of dense luminal structure, and C and D was predominantly localized to the tumor border (Supplementary Fig. 6b).

TAM populations were delineated in both the PDGfp and BrM datasets based on their expression of CD68, P2ry12, Iba1, CD206, and CD45 (Supplementary Fig. 5g). TAM_A cells showed higher overall expression of CD68 and CD45, resembling myeloid-derived macrophages (MDMs). TAM_B showed higher expression of Iba1, which may represent both activated MDMs and resident microglia. TAM_C showed the highest expression of P2ry12, which along with their localization outside of the tumor mass, suggests they are predominantly brain-resident microglia (Supplementary Fig. 6c). TAM_D cells showed the highest expression of CD206, localizing primarily to meningeal regions in similar distribution patterns to meningeal macrophages. The remaining immune cells that could not be reliably classified as known cell types were classified generally as CD45+, and represent a range of infiltrating myeloid cell types.

Astrocytes also clustered into two distinct patterns, with Astrocyte_A cells showing lower expression of GFAP and localizing to distant brain areas outside of the tumor, while Astrocyte_B cells showed higher expression of GFAP, and localized to the border regions of both PDGfp and BrM tumors (Supplementary Figs. 5h and 6d). Neurons stratified into two subtypes in PDGfp tumors based largely on their expression of FOXP1, which was specific to Neuron_B cells. FOXP1 was not included in the BrM antibody panel, and so Neuron_B cells were not identified in this dataset. Interestingly, blood vessels also separated into multiple categories in both PDGfp and BrM tumors, despite a lack of diverse phenotypic markers for the endothelium in the antibody panels we had developed (Supplementary Fig. 5i). Vessel_A cells mostly localized to brain regions outside of the tumor, while Vessel_B cells were associated with dysregulated vasculature within both PDGFp and BrM tumors. Vessel_C type cells were further stratified in PDGfp tumors based on high expression of αSMA (Supplementary Fig. 6c).

### Region and cell type quantification

**Fig. 5:**
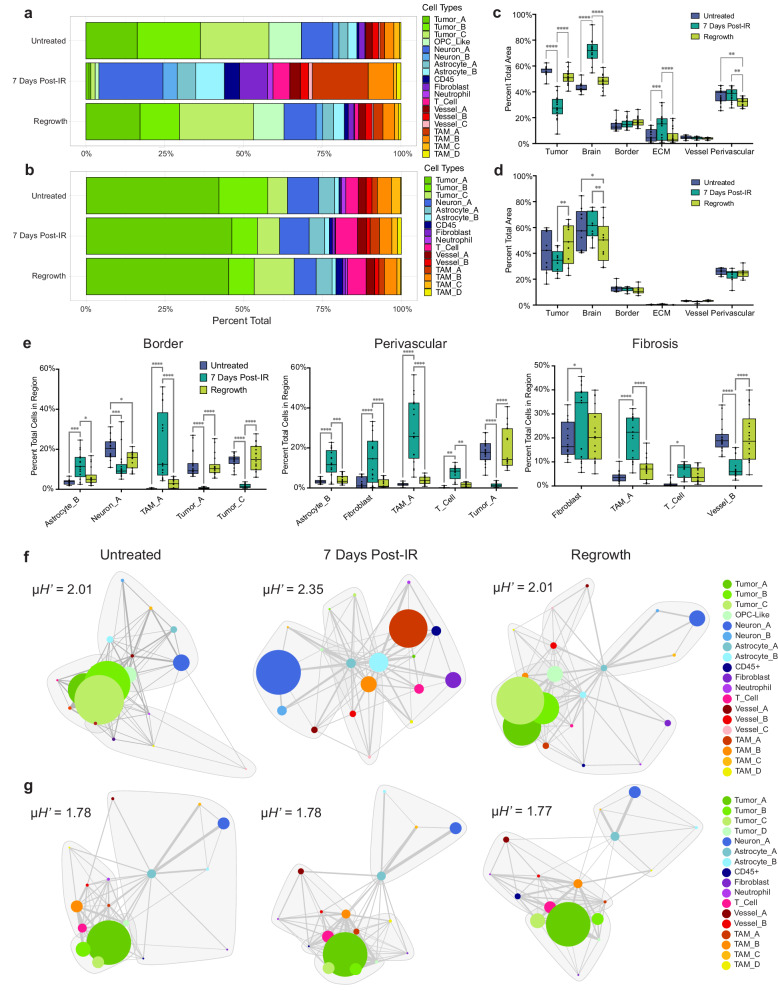
Spatial analysis of IR-treated brain tumors. Percentage of the total cellular composition quantified for each treatment group pooled across all images for (**a**) PDGfp and (**b**) BrM. Percentage of the area of tumor regions plotted for all groups for (**c**) PDGfp and (**d**) BrM. **e** Cell composition of tumor border, perivascular niches, and fibrotic regions for selected cell types in each treatment group. Box-plots for c-e show percent totals for each image (PDGfp Untreated *n* = 19 images, 7 days post-IR *n* = 17 images, Regrowth *n* = 18 images, BrM *n* = 9 images for each treatment). *p* values were calculated using two-way ANOVA test. *p* values for **c** (from left to right): <0.0001, <0.0001, <0.0001, <0.0001, 0.0002, <0.0001, 0.0067, 0.0045. *p* values for **d** (from left to right): 0.0023, 0.0317, 0.0044. *p* values for **e** (from left to right): Border, 0.0002, 0.0256, 0.0002, 0.0411, <0.0001, <0.0001, <0.0001, <0.0001, <0.0001, <0.0001, Perivascular < 0.0001, 0.0003, <0.0001, <0.0001, <0.0001, <0.0001, 0.001, 0.0094, <0.0001, <0.0001, Fibrosis, 0.014, <0.0001, <0.0001, 0.0486, <0.0001, <0.0001. Cell adjacency graph network plots of pooled treatment types and associated mean Shannon Diversity Index (*μH’*) for (**f**) PDGfp and (**g**) BrM microenvironments. Node sizes represent the binned range of percent-total cell populations, edge length is the mean distance between nearest neighbors, and edge width is the inverse standard deviation of mean distance. Gray outlines show clusters of nodes with similar neighbor relationships. Source data are provided as a Source data file.

Quantification of PDGfp tumors across treatment types revealed pronounced changes in the cellular landscape of glioblastoma samples following IR therapy (Fig. 5a). Untreated gliomas were predominantly comprised of tumor cell types, neurons, and astrocytes, with a mixed population of TAM cell types, and very small populations of T cells and neutrophils. PDGfp tumors 7-days post-IR showed the expected depletion of tumor cell populations, and a notable increase in TAM_A, TAM_B, T cell, neutrophil, and fibroblast populations. TAM populations more than tripled in percent-total, with marked increases in the TAM_A population in particular. Regrowth tumors largely recapitulated the cellular landscape of untreated tumors, with alterations in tumor type percentages, but with an increase in T cells compared to untreated tumors. Conversely, BrM tumors showed no widespread alterations in cellular landscape following treatment, or upon tumor regrowth, other than an increase in T cell populations (Fig. 5b).

Intratumoral regions were also significantly altered by IR in PDGfp tumors. There was a substantial increase in the percent-total of ECM-rich fibrotic regions following treatment, which was subsequently reduced upon tumor regrowth (Fig. 5c). This correlates with a similar increase in the percentage of fibroblasts 7 days post-IR. Annotated structural regions in BrM tumors were much more consistent following IR treatment, with significant differences in only the ratio of tumor area to brain, consistent with the observations of reduced tumor volume following treatment, and subsequent increases at the point of regrowth (Fig. 5d).

We further assessed the cellular composition of each of the annotated structural regions in both PDGfp and BrM tumors at each treatment point (Supplementary Fig. 7a–e). There were significant increases in TAMs, T cells, and fibroblasts in Border, ECM, and Perivascular regions of PDGfp tumors, which correlates with similar increases in areas of fibrosis (Fig. 5e). Conversely, BrM tumors only showed significant alterations in the distribution of tumor types and T cells in response to treatment (Supplementary Fig. 7d, e).

### Graph-based spatial network analysis

To analyze spatial relationships of cell types in HIFI data we employed orthogonal graph network and clustering-based approaches. We used graph-based network analysis to directly assess and summarize consistent cellular distance relationships from combined spatial data for each treatment type (Fig. 5f, g). Proximity network graphs used the Fruchterman-Reingold algorithm to set edge lengths between nodes as the mean distance between that cell type and the nearest neighbor of an alternate cell type. Edge weight was set as the inverse of standard deviation to reveal which interactions are more highly conserved between images. Edge weights that fell below a preset threshold were removed from the network. Node size for each cell type shows the relative percentage of those cells in the total population, binned into discrete node sizes. Network graphs were then clustered into sub-domains based on similar nodal connections.

Network plots for PDGfp tumors revealed consistent cellular relationships within and between treatment types, along with pronounced changes between tumor and immune cell populations following IR. Specifically, network plots revealed an increased association between surviving Tumor_A cells after treatment and increased populations of TAM_A, Fibroblasts, and Astrocyte_B cells. This correlates with the observed increase in fibrosis, and indicates a potential survival niche for radioresistant tumor cells (Fig. 5c, e). Additionally, these plots show the global extent of organized patterning, with 7 days post-IR samples having far less architectural structure and compartmentalization compared to untreated tumors. This was further reflected by cellular heterogeneity of each network; 2.35 mean Shannon Diversity Index for 7 days post-IR, versus 2.01 for both untreated and regrowth samples (Fig. 5f).

Consistent with the results discussed above, BrM network organization did not change substantially following treatment, with similar cellular distance relationships, and clear cellular compartmentalization depicting the tumor, surrounding brain, and tumor-brain border (Fig. 5g). Cellular diversity was also not substantially different based on mean Shannon Diversity.

### Cell neighborhood analysis

**Fig. 6:**
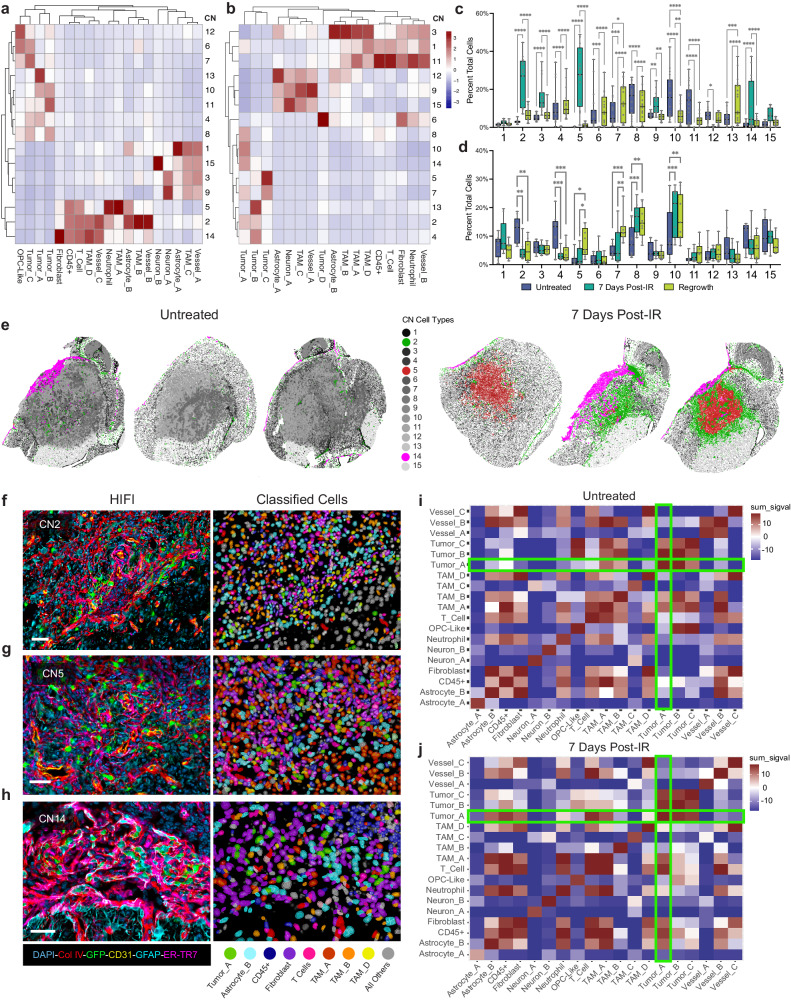
Differential Reorganization Response to IR Between Brain Tumor Models. Cellular composition column-scaled heatmaps of cell neighborhood (CN) analysis for (**a**) PDGfp and (**b**) BrM tumors. Percent total CNs across each treatment for (**c**) PDGfp and (**d**) BrM. Box-plots for c-d show percent totals for each image (PDGfp Untreated *n* = 19 images, 7 days post-IR *n* = 17 images, Regrowth *n* = 18 images, BrM *n* = 9 images for each treatment). *p* values were calculated using two-way ANOVA test. *p* values for **c** (from left to right): <0.0001, <0.0001, <0.0001, <0.0001, <0.0001, <0.0001, <0.0001, <0.0001, 0.0005, <0.0001, 0.0008, 0.0165, <0.0001, <0.0001, <0.0001, 0.0077, 0.0026, <0.0001, <0.0001, 0.008, <0.0001, <0.0001, 0.0116, 0.0003, <0.0001, <0.0001, <0.0001. *p* values for **d** (from left to right): 0.0013, 0.0014, 0.0003, 0.0008, 0.0122, 0.0471, 0.0006, 0.0079, 0.0003, 0.0027, 0.0004, 0.0052. **e** Representative positional plots for three untreated and three 7 days post-IR samples highlighting CNs 2, 5, and 14. **f**–**h** Representative images from 7 days post-IR samples showing HIFI (left) and digital pathology (right) images of CN2, CN5, and CN14 respectively. Scale bars = 40 µm. Heatmaps of cellular colocalization calculated as the sum of two one-tailed permutation test *p* values (sum_sigval) for (**i**) untreated samples and (**j**) 7 days post-IR samples. Green boxes indicate significant colocalization and anticorrelation for Tumor_A cells, for example. Source data are provided as a Source data file.

Cellular neighborhood analysis^38^ was performed with imcRtools^39^ by packaging HIFI data as SingleCellExperiment objects in R^40^. Treatment conditions were independently pooled for PDGfp and BrM datasets, and 15 cellular neighborhoods (CNs) were identified in each (Fig. 6a, b). Metadata for each cell was annotated with the CN they belonged to, and the percent-total of all CNs was quantified for each image in each treatment condition (Fig. 6c, d). The total proportion for 13 of 15 CNs was found to change significantly following treatment in PDGfp tumors, while only 4 CNs were significantly different between untreated and 7 days post-IR in BrM tumors. This again demonstrates the absence of spatial reorganization of BrM lesions in response to IR.

Of particular interest were the CN populations in PDGfp samples that were essentially unique to post-IR treated samples; CN2, CN5, and CN14. These 3 CNs clustered together in terms of cell type composition, being predominantly comprised of T cells, Fibroblasts, Astrocyte_B, Neutrophils, TAM_A, TAM_B, Vessel_B, Vessel_C, and small percentages of Tumor_A cells (Fig. 6a). Each of these CNs were mostly specific to regions of regressed lesions in samples 7 days post-IR treatment (Fig. 6e). Visual validation of regions enriched in these 3 CNs (Fig. 6f–h) correlate with fibrotic regions identified by machine-learning annotation (Fig. 5c).

Cell interaction analysis was performed to assess significant colocalization of cells within a 30 µm diameter in untreated and 7 days post-IR PDGfp tumors^39,41^. The Tumor_A cell type was found to be significantly anti-correlated with Fibroblast, T cell, Neutrophil, and Astrocyte_B cell types prior to treatment. 7 days post-IR treatment, Tumor_A cells became significantly colocalized with each of these cell types, as well as TAM_A, and CD45+ cells. (Fig. 6i, j). These results corroborate the previous orthogonal spatial analyses: each of the cell types cluster together in proximity network analysis of 7 days post-IR samples (Fig. 5f), they comprise CNs 2, 5, and 14 (Fig. 6a), and each are observed to be increased in the fibrotic ECM niche of treated samples (Fig. 5e, Supplementary Fig. 7c). The presence of the non-proliferative Tumor_A phenotype in this fibrotic niche suggests these spatial superstructures represent a survival niche for dormant radioresistant tumor cells (Watson, Zomer, [..], and Joyce, manuscript in revision).

The proximity network and cell neighborhood analyses of BrM tumors indicated that they do not respond to IR treatment in terms of spatial reorganization as PDGfp tumors do. Rather, the survival mechanism for IR-treated BrM samples appears to primarily rely on tumor cells entering into a quiescent state. Proliferating tumor cell populations are significantly reduced for all identified tumor types at 7 days post-IR (Supplementary Fig. 5f). Based on MRI volume data and image analysis, we did not observe significant reductions in tumor volume or cleaved-caspase 3+ apoptotic cells at the 7-day timepoint. These combined data indicate that BrM tumor resistance to IR therapy is driven predominantly by enrichment for lower proliferating cells, or by cell-state switching to quiescent states, rather than the formation of spatially protective niches. However, despite the difference in survival mechanisms, single dose focalized IR treatment was equally and transiently effective in BrM tumors as for PDGfp tumors.

## Discussion

High-dimensional digital pathology is a powerful approach for deriving biologically meaningful data from complex tissues, such as the multicellular TME. Our goal herein was to create a workflow that was fully agnostic to tissue processing, and required no special reagents, conjugated antibodies, or expensive equipment, so as to lower the barrier of entry to highly-multiplexed image analysis. By optimizing the workflow for a challenging ‘worst-case scenario’, in this case, lightly-fixed cryo-embedded glioblastoma and brain metastasis tissues, we have developed a robust imaging and analysis pipeline that is non-destructive for the tissue, allows for whole-slide image sizes, uses standard off-the-shelf antibodies, and that is scalable to high-throughput. This workflow includes the development of software tools that can handle large extracellular structural features, endogenous tissue autofluorescence, and densely packed irregular nuclei.

Some recent cyclic-IF approaches can now achieve between 60–100 markers^42^, which is especially useful for rare and precious patient samples where researchers seek to extract the maximum data possible. HIFI is envisioned as a platform to add to researchers’ options when dealing with samples and research questions that are not well addressed by other current techniques, and with the important components of ease of use and accessibility to a broad research community.

Combining large imaging areas, high magnification, and multiple markers of ECM components herein enabled a much broader environmental context for exploring cellular interactions in the TME. In addition to directly interrogating changes in fibrosis, desmoplasia, and healthy brain ECM, these markers facilitated improved classification of tumor domains by machine learning classifiers. Automated classifiers required minimal training due to the depth of our image data and provided valuable data points for spatially aware cellular analysis. These integrated metrics facilitated multiple orthogonal computational analyses to assess both the spatial organization of glioblastoma and brain metastasis models, as well as how that microenvironment organization responds to radiotherapy. We observed significant spatial reorganization in our murine glioblastoma model in response to IR, both in terms of cell landscape, spatial relationships, and superstructure patterning. Of particular interest were spatial niches that correlated with treatment-induced fibrosis specifically in glioblastoma, harboring quiescent tumor types. These niches indicated a potential survival mechanism whereby the local environment promotes tumor dormancy and survival, leading to subsequent glioblastoma recurrence. This is consistent with recent reports demonstrating ECM-mediated tumor cell dormancy^43^, and the identification of gene signatures related to fibrosis being predictive of more rapid tumor relapse and reduced overall survival in glioblastoma patients^44^.

Conversely, the breast-to-brain metastasis model showed no such organizational response to IR treatment. Instead, these tumors demonstrated reduced proliferation following treatment, with no significant debulking, or changes in cellular landscape other than increased T cells after treatment. Nonetheless, both glioblastoma and brain metastasis preclinical models received similar transient survival benefits from radiotherapy, indicating markedly distinct survival mechanisms between different tumor types in the same host tissue.

In summary, our HIFI approach is non-destructive, allows for large-scale imaging, and is adaptable for high-throughput analysis. Critically, the workflow reported herein is low-cost and opensource, such that it can be easily adoptable and broadly accessible for the scientific community. While we have demonstrated the use of the HIFI strategy in cancer tissues, this pipeline can equally be used in the analysis of any tissue type. We have purposely designed the workflow to be independent of the sample preparation, making it versatile for various types of tissue samples and eliminating the need for specialized reagents or equipment. Additionally, we have incorporated software tools to address the challenges of analyzing the complexity of the TME, such as identifying large extracellular structures and irregular nuclei. Our approach thus offers researchers a new strategy for studying the TME during tumor evolution, and following therapeutic intervention, enabling a deep and comprehensive interrogation of multicellular regional interactions.

## Methods

### Cell lines

Cell lines were cultured in DMEM + Glutamax (Gibco) containing 10% fetal bovine serum (FBS, Gibco) and 1% penicillin/ streptomycin (Gibco). DF1 chicken fibroblast and PyMT-BrM3 were grown under adherent conditions. The PyMT-BrM3 breast cell line was derived from the murine parental 99LN cell line, which was isolated from a metastatic lymph node lesion that arose in the MMTV-PyMT (murine mammary tumor virus; polyoma middle T antigen) breast cancer model (C57BL/6J background). This cell line was sequentially selected three times in vivo for brain-homing capacity, resulting in the PyMT-BrM3 variant used herein^5^. All cell lines were routinely authenticated for morphology and growth dynamics, and tested for mycoplasma contamination.

### Animal models, treatments, tissue processing

All animal studies were approved by the Institutional Animal Care and Use Committees of the University of Lausanne and Canton Vaud, Switzerland (License numbers: VD3804 and VD3688). Mice were housed in the Agora In Vivo Center (AIVC) animal facility in individually ventilated cages, under a 12 h light/dark schedule at 22 °C and in the presence of 2–4 cage mates. Standard autoclaved lab diet and water were provided ad libitum.

Murine genetically-engineered mouse models (GEMMs) of glioblastoma were generated as previously reported^27,45–47^. Nestin-Tv-a;*Ink4a/Arf*^*−/−*^ mice in the C57BL6 background were bred and maintained at the Agora Cancer Research Center, University of Lausanne (UNIL), Switzerland. At 5–6 weeks of age, glioblastomas were induced in GEMMs by injection of DF-1 cells producing viral vectors containing both PDGF-B and GFP as described previously^45^, and monitored biweekly by MRI for tumor development. Female C57BL6 mice received intracranial injection of the PyMT-BrM3 breast-to-brain metastasis cell line^29^ at matched age and cranial coordinates for tumor initiation in the PDGfp model. The maximum humane endpoint tumor size approved per out animal protocols was 1.5 cm^3^ for PDGfp and 0.2 cm^3^ for BrM, all mice were euthanized before reaching the maximum allowed tumor burden. Once tumors exceeded 20 mm^3^, mice were randomly assigned without blinding to treatment groups (untreated, 7 days post-IR, or rebound). Mice were anesthetized by isofluorane and administered a single whole-brain focalized dose of ionizing radiation using the Precision X-Ray X-RAD SmART irradiator (10 Gy for PDGfp mice, 15 Gy for BrM mice). MRI monitoring was continued following IR treatment, and mice were euthanized upon tumor recurrence as approved by the Institutional Animal Care and Use Committee.

Animals were euthanized via pentobarbital injection, and perfused intracardially with 10 ml of PBS, followed by 10 ml of PLP buffer. PLP buffer consisted of 1% paraformaldehyde (PFA), 0.2% NaIO4, 37.5% L-lysine and 37.5% P-buffer (containing 81% of Na_2_HPO_4_, 19% of NaH_2_PO_4_ diluted in water, pH = 7.4) (Sigma Aldrich). Brains were excised and fixed in PLP overnight at 4 °C with gentle shaking. Tissue samples were washed in PBS, then transferred to 30% sucrose overnight at 4 °C with gentle shaking. Finally, mouse brains were flash-embedded in Optimal Cutting Temperature (OCT) compound (Tissue-Tek), then cryosectioned onto Fisherbrand Superfrost Plus slides at 10 µm thickness. All slides were stored at −80 °C until used for staining and imaging experiments.

### Antibody labeling and imaging

Prior to study, all antibodies were validated for compatibility with the HIFI approach (Supplementary Note 1). Tissue section slides were thawed at room temperature (RT) and allowed to dry for 15 min, then OCT was removed in a PBS bath at RT for 5 min with gentle agitation. An optional step at this point is a post-fixation in 4% PFA on ice for 5 min, followed 2 × 5-min PBS washes and quenching in 0.1 M glycine for 20 min at RT. Slides were then placed into humidified chambers, and tissue sections were outlined with a hydrophobic barrier using peroxidase-antiperoxidase (PAP) pens. Tissue sections were permeabilized with 0.2% Triton X-100 in PBS for 10 min at RT, then washed twice in PBS at RT with gentle agitation. Slides were stained for nucleic acid with DAPI at 1:2000 dilution in HIFI Staining Buffer (HSB; 5% normal donkey serum (Merck) and 100 mM NH_4_Cl (Sigma Aldrich) in PBS) for 10 min at RT, then washed 3 times in PBS at RT with gentle agitation. SlowFade Diamond (Invitrogen) mounting medium and 22 ×22 cm glass coverslips were used to mount slides for imaging. All slides were imaged on a Zeiss Axio Scan Z1 with Colibri LED light source. The LED power was set to the lowest possible settings based on the photonic energy of each wavelength. The 350 channel was set to 5% power, 488 set to 20%, 555 set to 50%, 647 set to 50%, and 750 set to 100%. Exposure times were set to experimentally predetermined lengths to capture DAPI signal and endogenous tissue autofluorescence for all channels. Following imaging, slides were placed in a horizontal slide rack with the bottom removed, this rack was then placed into a PBS bath with gentle agitation until coverslips fell off without manipulation. Sections were blocked with HIFI Blocking Buffer (HBB; 10% normal donkey serum (Merck), 150 mM Maleimide (Sigma Aldrich), and 100 mM NH_4_Cl (Sigma Aldrich) in PBS) for 1 h at RT as previously described^26^. HBB was then removed and replaced with the primary antibody mix in HSB, and allowed to incubate for 1.5 h at RT on an orbital rocker. Slides were washed 3 times in PBS for 5 min at RT with gentle agitation following primary antibody incubation, then secondary antibody mix with conjugated fluorophores in HSB was added, and slides were incubated for 1 h at RT on an orbital rocker. All secondary antibodies were raised in donkey to optimize compatibility. Slides were washed 3 times in PBS for 5 min at RT with gentle agitation following secondary antibody incubation, and directly conjugated antibodies in HSB were added, and slides were incubated for 1 h at RT on an orbital rocker. If no conjugated antibodies were included in that round of antibodies, slides proceeded to the next step. Slides received three final washes in PBS for 5 min at RT with gentle agitation, and were then mounted for imaging. Following imaging of the first round of markers, coverslips were again removed, and antibodies were eluted by adding elution buffer (0.5 M Glycine, 3 M guanidine hydrochloride (Sigma Aldrich), 3 M Urea (Sigma Aldrich), 40 mM tris(2-carboxyethyl)phosphine (Sigma Aldrich), in deionized H_2_O) for 3 minutes with gentle agitation at RT. The above process was repeated for all rounds of antibody panels. HIFI staining was performed in two separate rounds of imaging, first for half of PDGfp samples (PDGfp1), then for the other half of PDGfp samples (PDGfp2) and all BrM samples. PDGfp1, PDGfp2, and BrM were each analyzed and clustered independently to avoid batch effects. PDGfp1 and PDGfp2 datasets were then merged at the point of cellular annotation.

Image post-processing was performed for all 16-bit tile-scanned images using the Zeiss Zen software platform. Tile stitching and fusion was performed for all images. Background subtraction was performed with the rolling-ball subtraction method using a diameter of 75 µm.

### Image alignment, cell detection, and cell classification

The alignment script was developed in Python to reassemble images from multiple imaging rounds into full-resolution OME TIFF images. For each HIFI image, the DAPI channel of the first imaging round was loaded to serve as the alignment reference. Subsequent DAPI channels were loaded in sequence. Rigid body (or affine) transformation aligned each DAPI channel to the reference by minimizing the squared difference between the reference and the transformed channel using PyStackReg^31^. The transform was then applied to each image channel of that round, keeping only one channel in memory at a time. The transformed round was then saved to disk, and the next round was processed. Aligned channels from all rounds were concatenated into a single OME TIFF and compressed into a pyramidal image format. Metadata for pixel scale and protein marker identifiers of each channel were propagated to the final image.

Cell segmentation was performed using the CNN-based StarDist algorithm^33^ implemented in Qupath. For improved accuracy, a model for deep learning segmentation was generated using manually segmented training data from the same image data set analyzed in this study. Images of 18 tissue samples were acquired which were derived from various healthy organs and tumors from different mouse models. Image regions, with on average 305 cells per image, were manually selected to capture the wide range of variation in cellular appearance. The images were down-sampled from 2048 × 2048 to 512 × 512 for accommodating the receptive field of StarDist. Manual annotations were performed using QuPath v0.3.x by eight volunteer microscopists, who were assigned one image from each of the 18 tissue samples. The results in geojson annotation files were converted to label maps whereby overlaps were resolved by generating a distance map for each cell and assigning pixels to a cell only when its value in the distance map was greater than for all other cells. This resulted in a manually annotated dataset of 144 images. This dataset was augmented by adding publicly available datasets including BBBC020, BBBC038v1, and BBBC039v1 from the Broad Bioimage Benchmark Collection^48–50^. For BBBC038v1 we used the fluorescence images of “stage1 train” only, from the unofficial fixes by Konstantin Lopuhin (https://github.com/lopuhin/kaggle-dsbowl-2018-dataset-fixes). We also used the images from Coelho et al., which consists of hand-segmented nuclear images of 3T3 and U20S cells^51^.

A probability score was generated for each nucleus predicted by StarDist to indicate nuclear segmentation confidence, and used for quality control checks. Each nucleus was expanded by 2.5 µm to approximate the surrounding cytoplasm. The expansion was constrained by the size of the detected nuclei, so that a cell was not larger than 1.5 times the size of its nucleus. This produced four measurement zones per object; nucleus only, cytoplasm only, whole cell, and cell membrane. The following measurements were taken for every single-cell object: X-Y object centroid coordinates of each nucleus, area, perimeter, and circularity of each nucleus and cell, distance of each object centroid to the nearest regional annotation border, and distance of each object centroid to the nearest neighbor object of an alternate cell type. For subsequent analyses, either the nuclear MFI or the cell MFI were used, depending on the expression of the marker (Supplementary Table 2).

Semi-supervised annotation was performed using FlowSOM, a tool that leverages self-organizing maps to interpret multiplexed flow cytometry data^35^. Self-organizing maps are artificial neural networks that perform unsupervised dimensionality reduction while maintaining the topological structure. We analyzed the two PDGfp batches and all BrM tumors separately, by building a self-organizing map with 10 × 10 grid dimensions, for a total of 100 nodes. Input data was the mean MFI intensity of marker proteins (nuclear or whole-cell according to the biology) scaled to a 0-1 range, with the highest values clipped to the 99.7^th^ percentile. The assignment of nodes to clusters was guided by: (i) the automatic optimized metaclustering by flowSOM on the minimal spanning tree, (ii) the heatmap of mean MFI intensities for marker proteins in each node, (iii) the mapping of node annotations to QuPath objects and visual inspection of marker intensities. The annotation of clusters was defined across batches and mouse models based on the heatmap of the expression of marker proteins in clusters and visual inspection of cluster annotations in QuPath (Supplementary Fig. 4a–c).

### Machine learning object annotation

Regional tissue annotation was performed in QuPath^32^ (version 0.4.4). Binary classifiers were generated to create ROIs for the entire lesion, vessels, areas of ECM, and tumor versus surrounding parenchyma. All classifiers used artificial neural network (ANN) machine learning, and were trained off of 25% of image data. Two derivative annotations were created via modifications of the above ROIs; the tumor border, and the perivascular niche. The lesion classifier was trained using all available channels, holes and fragments less than 20,000 pixels were removed from ROIs, and the region was expanded 750 µm into the parenchyma to capture adjacent brain tissue. Manual removal of tissue deformations resulting from tissue sectioning or misalignment was performed on all lesion ROIs. Vessel classifiers were trained using only CD31, VE-cadherin, and CD13 channels, holes and fragments less than 20 pixels were removed. Vessel ROIs were expanded by 15 µm to create perivascular annotations. The tumor versus brain classifier used for PDGfp samples was created using the DAPI, GFP, Sox9, Sox2, Olig2, NeuN, NF-H, CSPG5, WGA, and Lamin AC channels. The tumor versus brain classifier used for BrM samples was created using DAPI, E-cadherin, EpCAM, CK14, CK8, NeuN, and NF-H. Holes and fragments less than 4000 pixels were removed. The intersection between the tumor and brain was expanded by 70 µm to create the tumor border annotation. The ECM classifier was trained using the Col I, Col IV, CD13, WGA, CSPG5, Laminin, ERTR7, and Fibronectin channels. Holes less than 50 and fragments less than 300 pixels were removed from ROIs.

### Spatial analysis

Cell neighborhood and interaction analysis were performed in R with RStudio using the imcRtools^39^ package version 1.0.2, and nearest neighbor distance network analysis used the igraph^52^ package. HIFI image measurements were repackaged into SingleCellExperiment objects for analysis. An expansion radius of 30 µm was used to generate a spatial connectivity map of each image, connectivity maps for all images were pooled together and clustered to identify 15 distinct cell neighborhoods. Cell object identifiers were reassigned with their respective cluster number and quantified for each image and treatment type. Interaction analysis was performed using the ‘classical’ method, randomly reassigning each cell type with 1000 iterations to create a null distribution pattern for the statistical comparison of significant cellular interactions.

Mean nearest neighbor distance measurements for each treatment type were compiled into distance matrices of mean and standard deviation, and distance networks were generated with igraph using the Fruchterman–Reingold layout algorithm to set edge lengths as proportional to the mean distance between two cell types. Edge widths were modified based on the standard deviation of the mean, such that edge width was the inverse of variability (1/SD). This was then multiplied by a constant esthetic value (100) for visualization of edge width heterogeneity. Edge widths below a threshold were removed from each network. The edge width threshold was determined as the median standard deviation of a network multiplied by an experientially predetermined network heterogeneity cofactor constant (0.8) so that the network represents inherent heterogeneity while being comparable to other networks in the experiment. Node size for each cell type was based on binned percent-total populations, so that cell type from <1% to >25% of the total population corresponded to node sizes between 3 and 70 respectively. Network plots were then clustered (gray outlines) using Louvain clustering with a resolution of 1.5 to determine cell type nodes with similar distance patterns.

### Statistics and reproducibility

All relevant experiments and analyses had a minimum of 3 independent repetitions, and each dataset presented in the study had at least 3 technical replicates included. Statistical analyses and Shannon Diversity Index scores were performed in R with RStudio, or in GraphPad Prism (GraphPad 9.0 software). Two-way ANOVA tests were used for all between-group comparisons, with the level of significance defined as *P* < 0.05. Figure asterisks correlate to *P*-value thresholds: ns > 0.05, *≤0.05, **≤0.01, ***≤ 0.001, ****≤0.0001.

### Reporting summary

Further information on research design is available in the Nature Portfolio Reporting Summary linked to this article.

### Supplementary information

Supplementary Information

Reporting Summary

Peer Review File

### Source data

Source Data

## Data Availability

The StarDist cell segmentation model and associated training data are available from the GitHub repository of the iMAXT consortium: https://github.com/TristanWhitmarsh/IMAXT_StarDist_Cellpose. All other data are available from the following Zenodo repository, DOI: 10.5281/zenodo.10778429. The file sizes of primary HIFI image data exceed hosting capacity, and so are available upon request. All remaining data can be found in the Article, Supplementary and Source data files. Source data are provided with this paper.

## Code availability

The HIFI Alignment Tool is available from the GitHub repository of the Jean Hausser lab: https://github.com/jhausserlab/HiFiAlignmentTool. All other code is available from the following Zenodo repository, 10.5281/zenodo.10778429.
