# Supplementary material for: Microenvironmental reorganization in brain tumors following radiotherapy and recurrence revealed by hyperplexed immunofluorescence imaging
Source: Nat Commun. 2024 Apr 15;15:3226. doi: 10.1038/s41467-024-47185-9 (PMC11018859; doi:10.1038/s41467-024-47185-9)
Supplement: Supplementary file 1 — Supplementary Information [file 41467_2024_47185_MOESM1_ESM.pdf]

Supplementary Figure 1

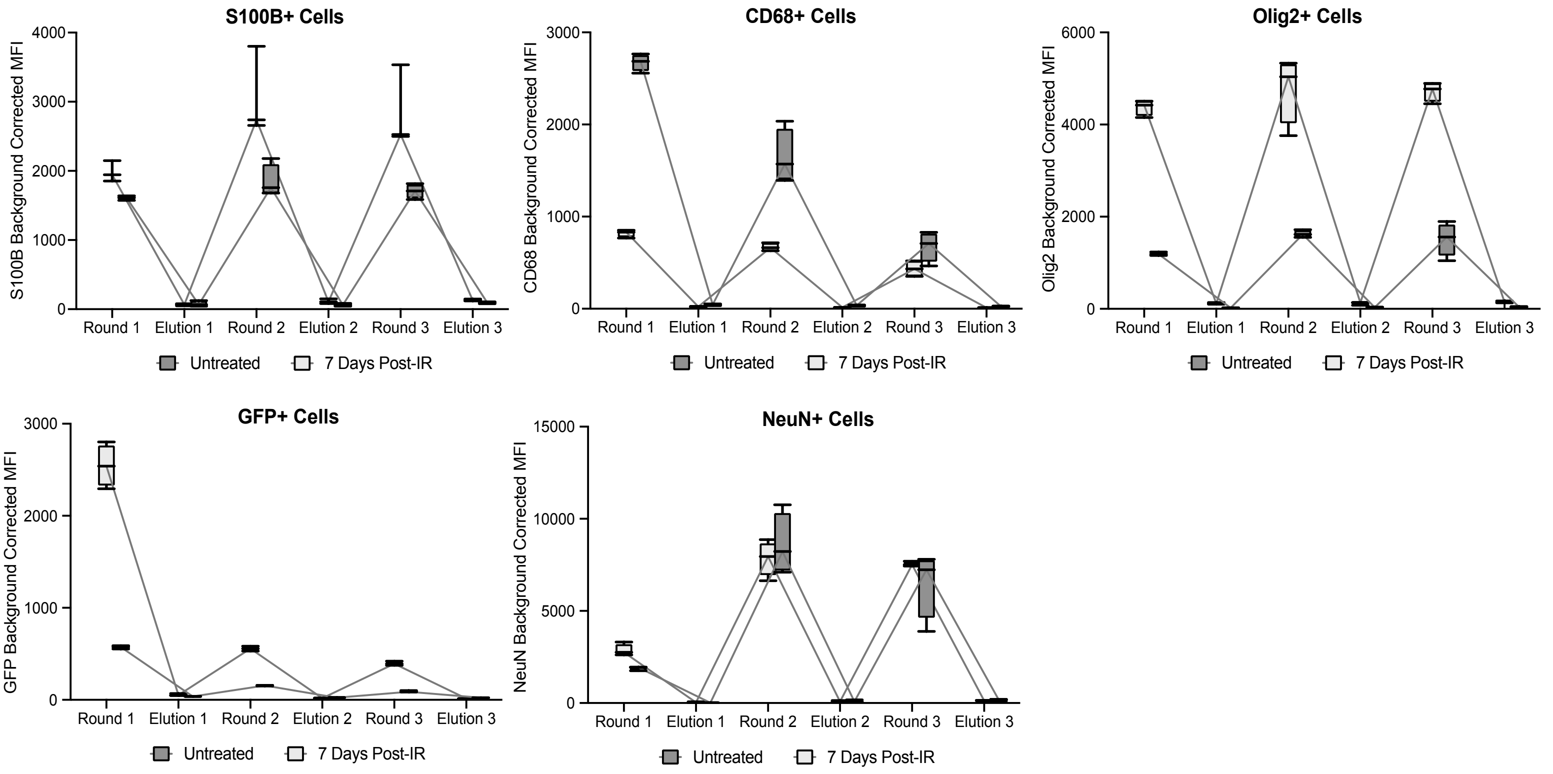

Supplementary Figure 1. Antibody Panel Design and Validation. Single-cell mean fluorescence intensity (MFI) for untreated and 7 days post-IR PDGfp sections (n=4 mice for each) imaged after primary and secondary antibody labeling, and after elution and secondary antibody labeling. Imaging scheme was repeated 3 times for each marker. Plots show mean MFI of marker-positive cells for each image, minus the mean MFI of marker-negative cells to correct for background. Source data are provided as a Source data file.

Supplementary Figure 2

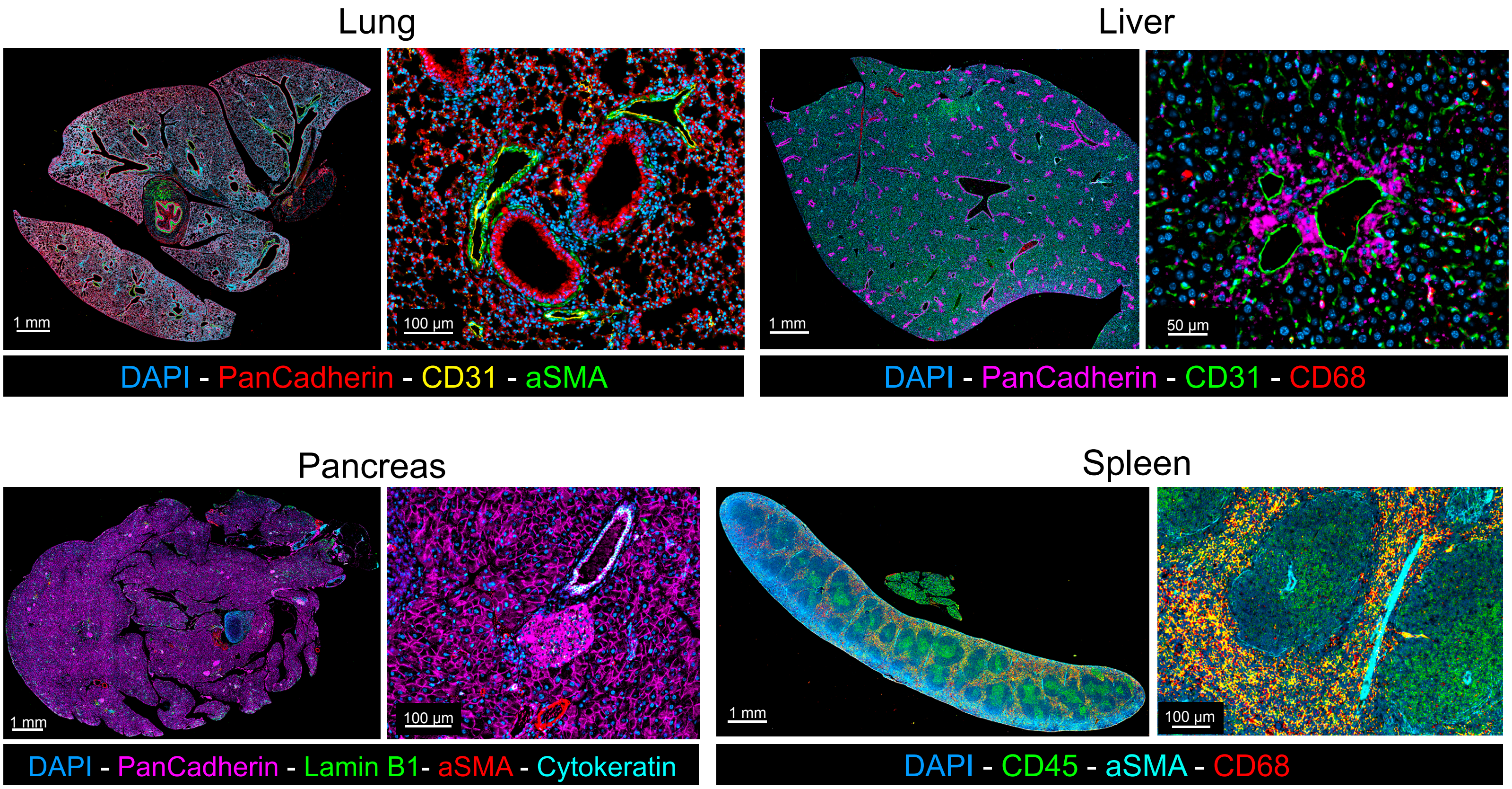

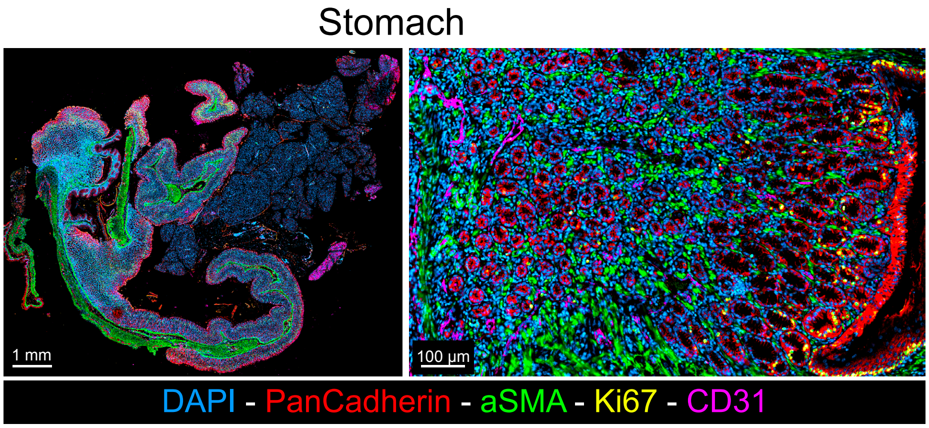

Antibody Panel		
CD3	CD31	ERTR7
CD68	Ki67	Col IV
CD45	PanCadherin	Pan-cytokeratin
Lamin AC	Vimentin	aSMA

Supplementary Figure 2. HIFI Compatibility with FFPE Tissues. Representative HIFI images of murine FFPE tissue samples from the lung, liver, pancreas, spleen, and stomach. Dewaxing and pH 9.0 EDTA antigen retrieval were performed on all tissue sections. HIFI was used to label all sections with a 3-round antibody panel (lower right panel).

Supplementary Figure 3

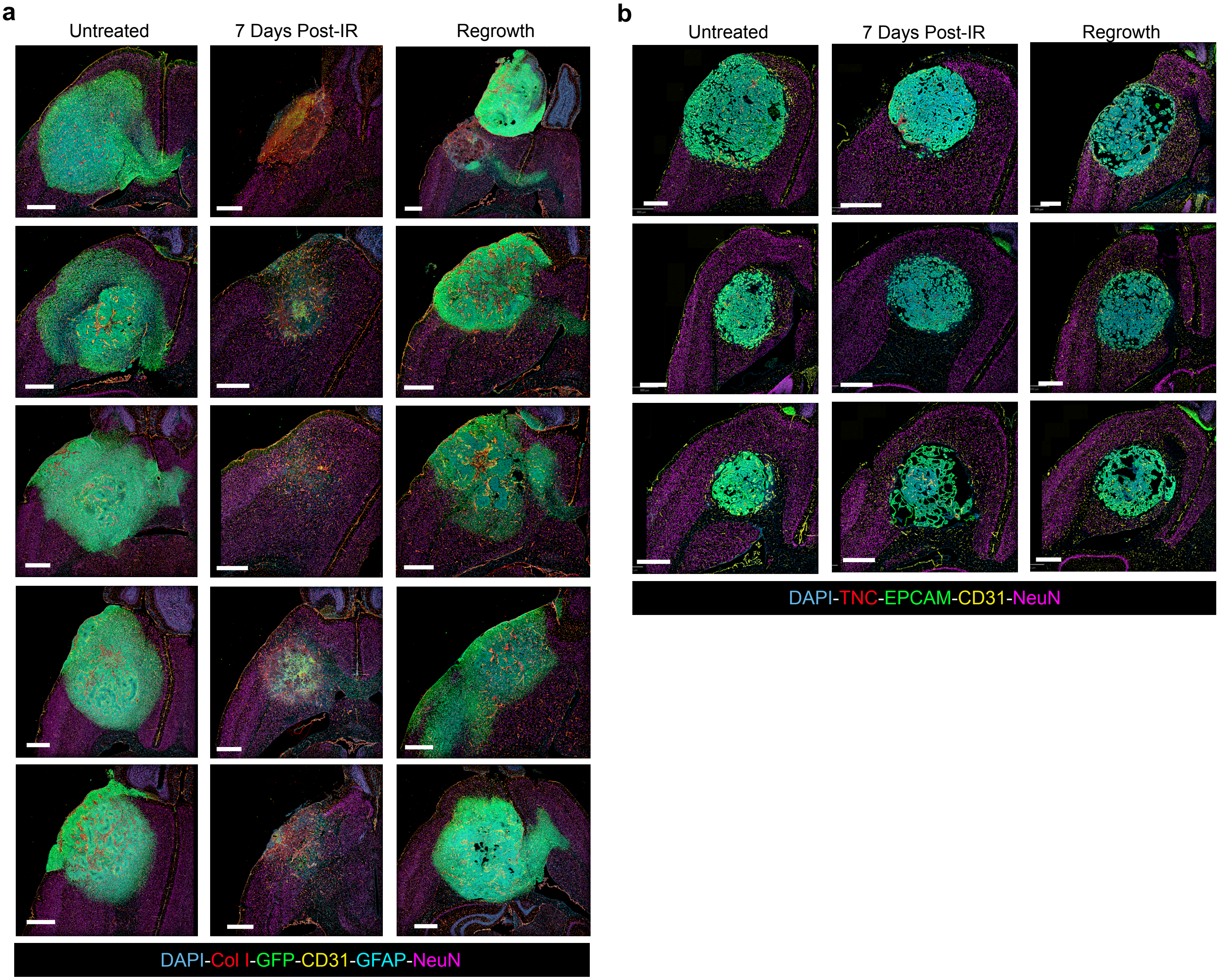

Supplementary Figure 3. HIFI Images for PDGfp and BrM Sample Sets. Representative images from each murine sample in each treatment category for **(a)** PDGfp and **(b)** BrM. Scale bars = 800 µm.

Supplementary Figure 4

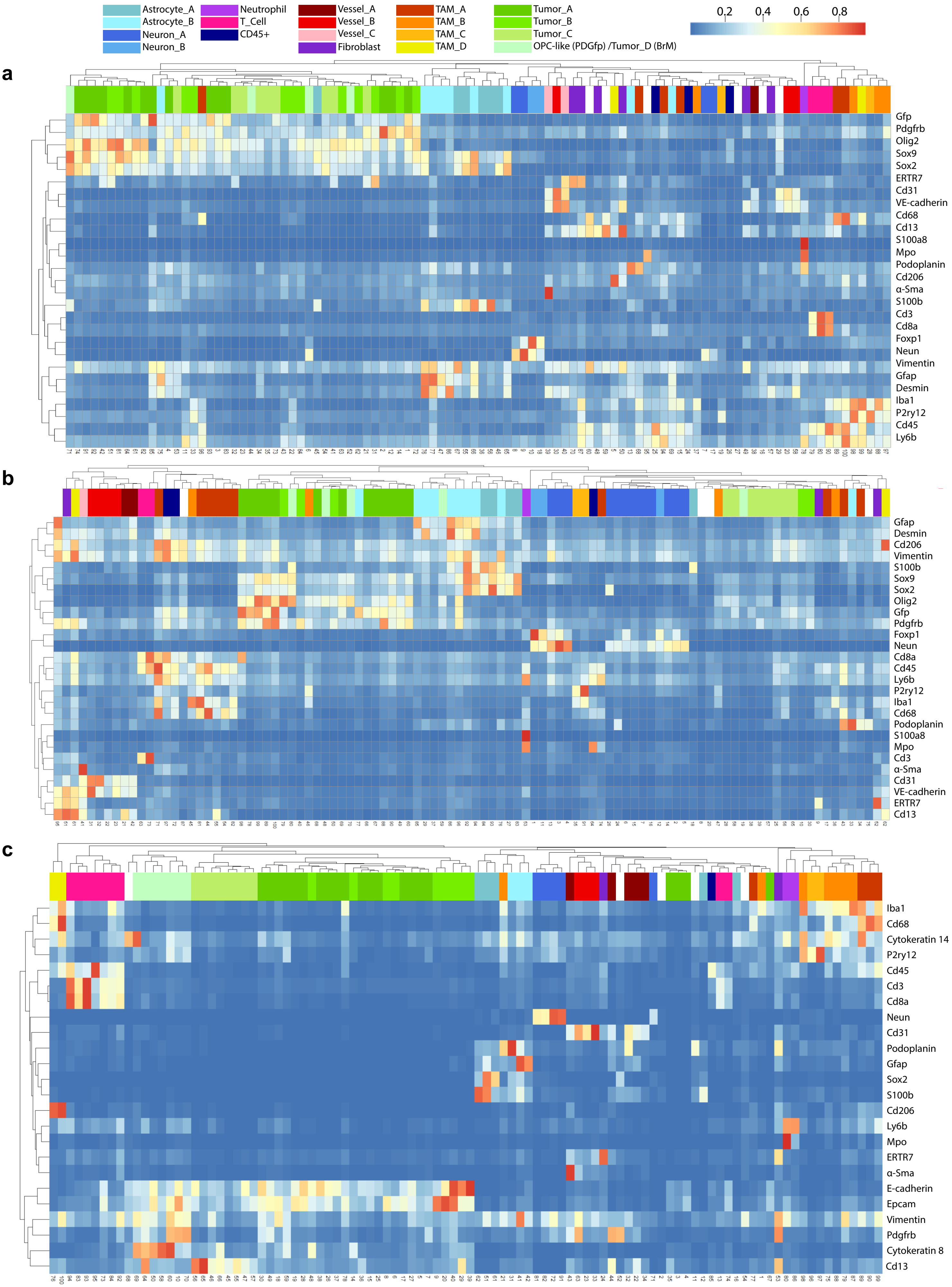

Supplementary Figure 4. Delineating Multiple Cell Phenotypes. Heatmaps of mean scaled mean fluorescence intensity from FlowSOM node clustering of **(a)** batch 1 PDGfp HIFI imaging (n=30 images), **(b)** batch 2 PDGfp imaging (n=24 images), and **(c)** BrM imaging (n=27 images). Each node cluster is annotated with the corresponding cell type assignment.

Supplementary Figure 5

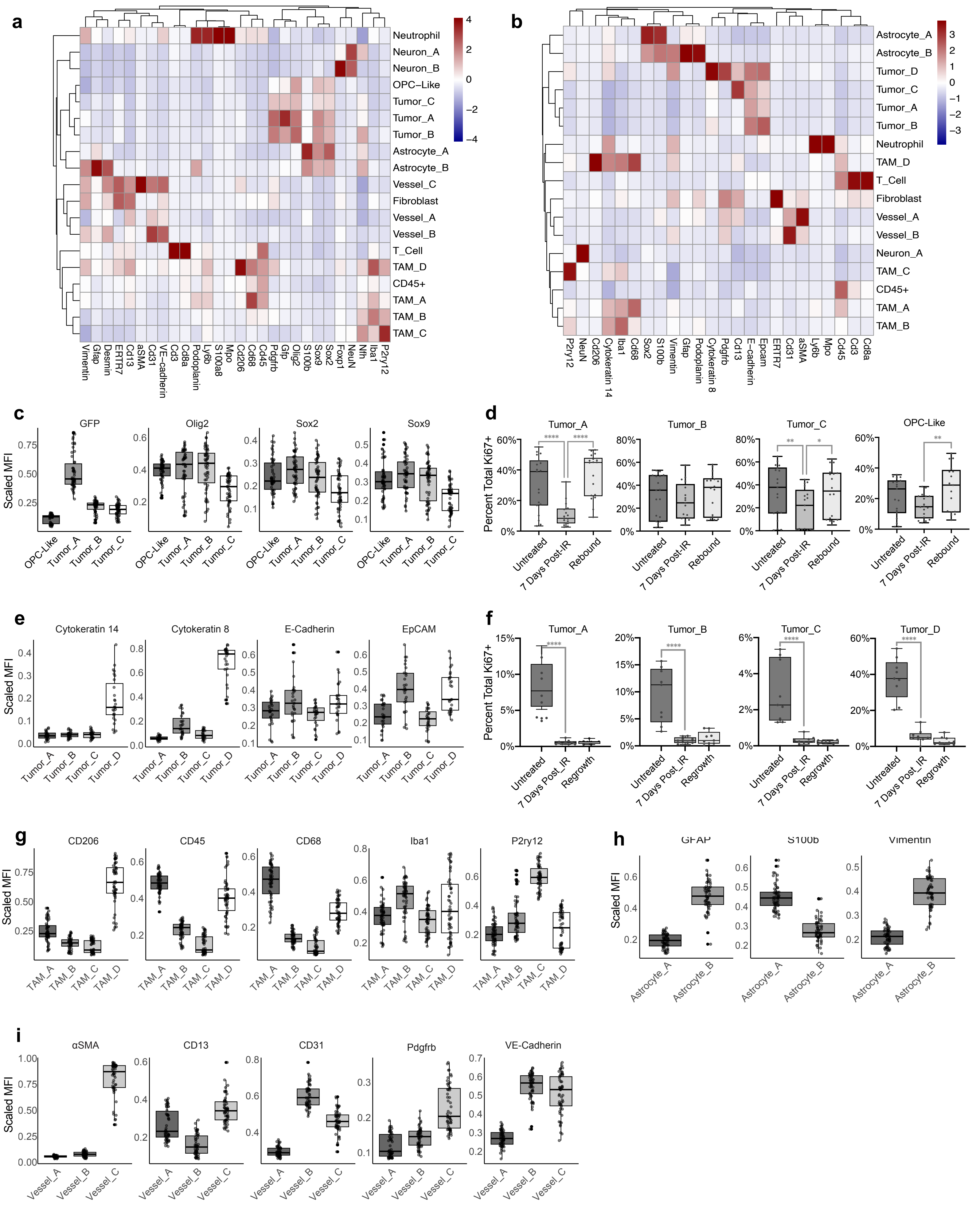

Supplementary Figure 5. Cell Phenotype Stratification. Heatmaps of mean scaled marker expression from cell type clusters identified in **(a)** PDGfp and **(b)** BrM samples. **(c)** Scaled mean fluorescence intensity (MFI) for markers delineating Tumor phenotypes in PDGfp samples (n=54 images). **(d)** Percent total Ki67-positive cells of each PDGfp Tumor cluster in each treatment condition. **(e)** Scaled MFI for markers delineating Tumor phenotypes in BrM samples (n=27 images). **(f)** Percent total Ki67-positive cells of each BrM Tumor cluster in each treatment condition. **(g)** Scaled MFI for markers delineating TAM phenotypes. **(h)** Scaled MFI for markers delineating Astrocyte phenotypes (n=54 images). **(i)** Scaled MFI for markers delineating Vessel phenotypes (n=54 images). Box-plots for d and f show percent totals for each image (PDGfp Untreated n=19 images, 7 days post-IR n=17 images, Regrowth n=18 images, BrM n=9 images for each treatment). p values were calculated using two-way ANOVA test. Figure asterisks correlate to p value thresholds: *< 0.05, **< 0.01, ***< 0.001, ****< 0.0001. Full plot metric are provided in Source Data file.

Supplementary Figure 6

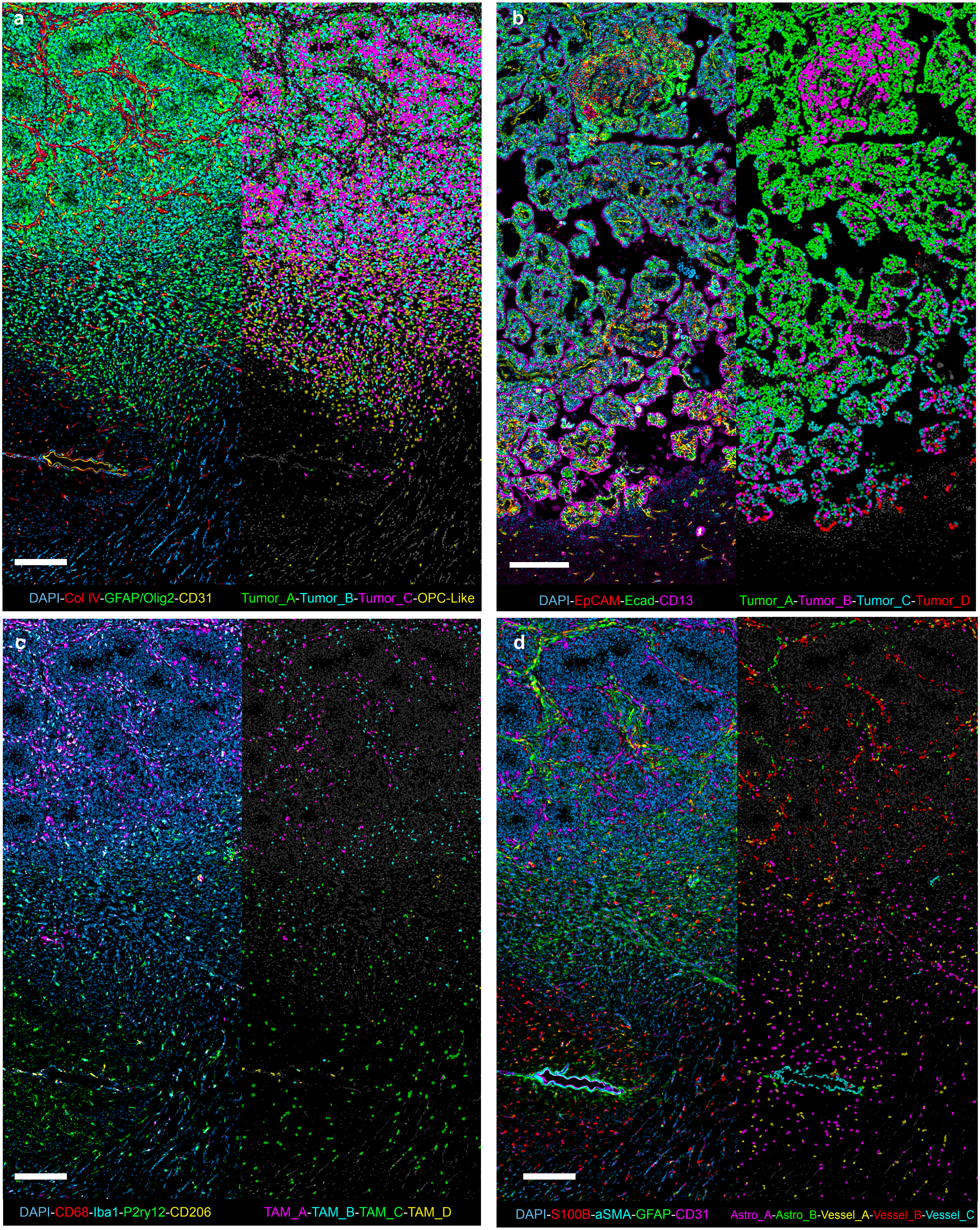

Supplementary Figure 6. Spatial Validation of Cell Phenotypes. Representative images showing spatial patterning of **(a)** PDGfp tumor clusters, **(b)** BrM tumor clusters, **(c)** TAM clusters, and **(d)** Astrocyte and Vessel clusters. Each panel depicts the HIFI image (left) and corresponding annotated digital pathology image (right). Scale bars = 200 μm.

Supplementary Figure 7

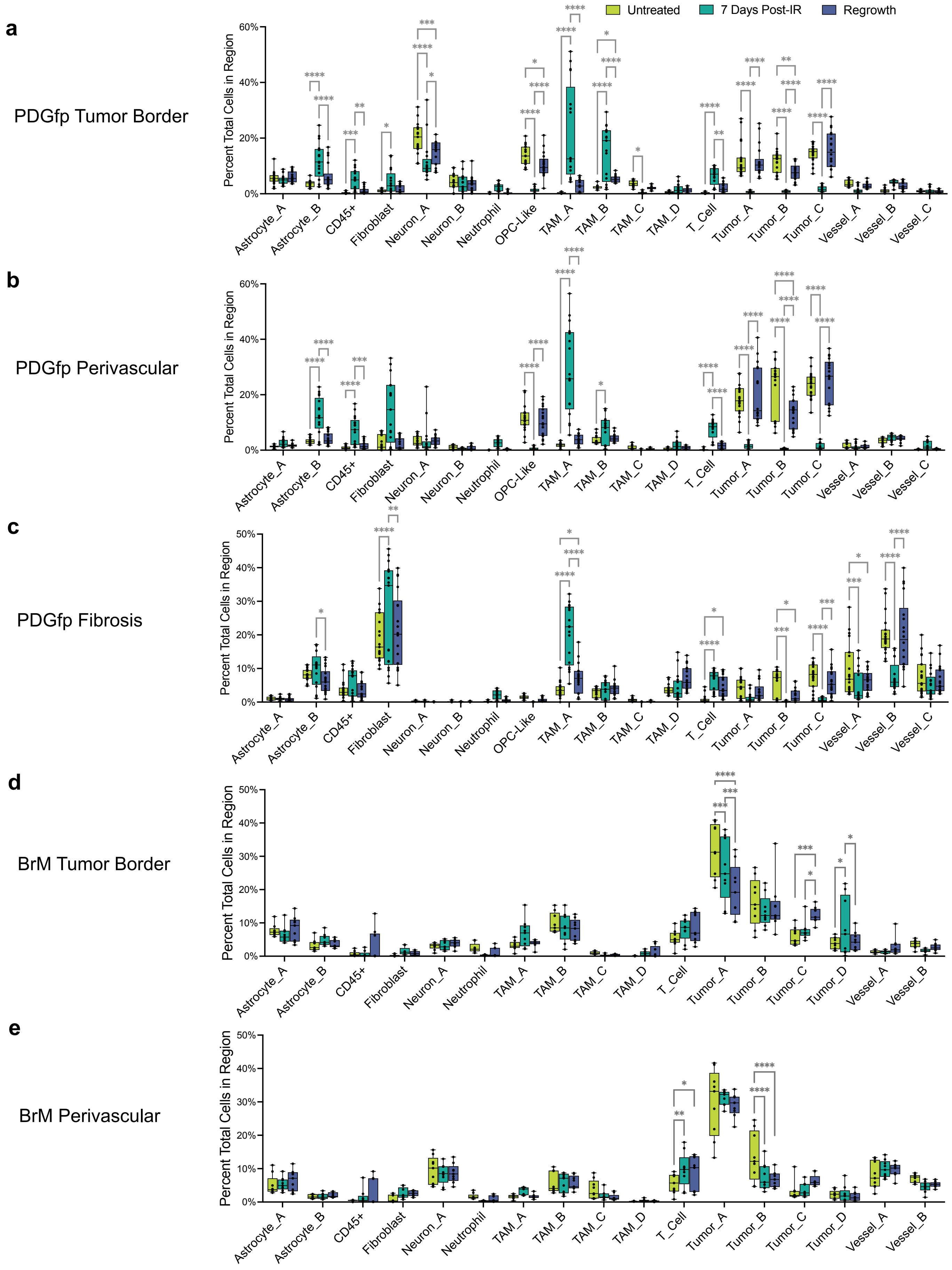

Supplementary Figure 7. Cellular Composition of Regional Tumor Features. (a-c) Cell type percent-total composition of PDGfp tumor border, perivascular regions, and ECM fibrosis, respectively. **(d, e)** Cell type percent-total composition of BrM tumor border, and perivascular regions, respectively. All box-plots show percent totals for each image (PDGfp Untreated n=19 images, 7 days post-IR n=17 images, Regrowth n=18 images, BrM n=9 images for each treatment). p values were calculated using two-way ANOVA test. Figure asterisks correlate to p value thresholds: *< 0.05, **< 0.01, ***< 0.001, ****< 0.0001. Source data are provided as a Source data file.

Supplementary Table 1

Tumor	
Cytokeratin 14	Basal epithelial
Cytokeratin 8	Luminal epithelial
E-cadherin	Epithelial
Epcam	Tumor
Immune	
Cd3	Pan T cell
Cd8a	Cytotoxic T cell
Iba1	Macrophage
Cd68	Macrophage
P2ry12	Microglia
Cd45	Pan leukocyte
Cd206	TAM activation
Ly6b	Neutrophil
Mpo	Neutrophil activation
ECM	
Tenascin-C	Fibrotic ECM
Neurons	
FoxP1	Neural stem cell
NeuN	Pan neuronal
Nf-H	Neurofilament
Vascular	
ER-TR7	Reticular fibroblast
Pdgfrb	Pan perivascular cells
Cd31	Endothelial
αSma	Mural cells
Cd13	Pericyte
Cell State	
Ki67	Proliferation
Cleaved Caspase-3	Apoptosis
Hif-1α	Hypoxia
Lamin AC	Nuclear envelope
Glial	
Vimentin	Astrocyte subset
Gfap	Astrocyte activation
Podoplanin	Reactive gliosis
S100b	Pan astrocyte

Supplementary Table 1. BrM HIFI Marker Panel. List of multiplexed markers used specifically for BrM samples, indicating category of marker, marker name, and marker target.

Supplementary Table 2

Marker	Species	Round (PDGfp)	Round (BrM)	Dilution	Conjugation	Channel	Clone	Supplier	Measurement Area	For Classification	Catalog	RRID
DAPI	NA	0	0	1/2000	NA	350	NA	Thermo Fischer	Nucleus	No	D1306	NA
CD3	Rat	1	1	1/50	NA	750	KT3	Abcam	Cell	Yes	AB33429	AB_726330
CD8a	Rat	1	1	1/50	647	647	53-6.7	BD Pharmingen	Cell	Yes	557682	AB_396792
Iba1	Rabbit	1	1	1/400	NA	555	Polyclonal	Wako	Nucleus	Yes	019-19741	AB_839504
GFP	Chick	1		1/500	NA	488	Polyclonal	Abcam	Cell	Yes	AB13970	AB_300798
CD68	Rat	2	2	1/100	NA	750	FA-11	Bio-Rad	Cell	Yes	MCA1957	AB_322219
P2RY12	Rabbit	2	2	1/200	NA	647	Polyclonal	Anawa	Nucleus	Yes	AS-55043A	NA
CD45	Rat	2	2	1/30	PE	555	30-F11	BioLegend	Cell	Yes	103106	AB_312971
CD206	Goat	2	2	1/200	NA	488	Polyclonal	R&D Systems	Cell	Yes	AF2535	AB_2063012
FoxP1	Rabbit	3		1/100	NA	750	Polyclonal	Novus Biologicals	Nucleus	Yes	NBP1-89410	AB_11023624
Ly6b	Rat	3	3	1/50	NA	647	7/4	BioRad	Cell	Yes	MCA771A	AB_1102791
HIF1a	Goat	3	3	1/200	NA	555	Polyclonal	Abcam	Cell	No	ab16066	AB_302234
Vimentin	Chick	3	3	1/300	NA	488	Polyclonal	Abcam	Nucleus	Yes	ab24525	AB_778824
Olig2	Goat	4		1/100	NA	750	Polyclonal	R&D Systems	Cell	Yes	AF2418	AB_2157554
Ki67	Rat	4	4	1/50	NA	647	SolA15	Thermo Fisher Scientific	Nucleus	No	17-5698-82	AB_2688057
Sox9	Rabbit	4		1/100	NA	555	EPR14335-78	Abcam	Nucleus	Yes	ab225541	AB_3073665
ERTR7	Rat	5	5	1/400	NA	750	Polyclonal	BioTechne	Cell	Yes	NB100-64932	AB_963381
Sox2	Goat	5	5	1/200	NA	647	Polyclonal	R&D Systems	Nucleus	Yes	AF2018	AB_355110
PDGFRB	Rabbit	5	5	1/300	NA	555	Y92	Abcam	Cell	Yes	ab32570	AB_777165
Lamin AC	Chick	5	5	1/150	NA	488	Polyclonal	Antibodies.com	Cell	No	A85443	AB_2748991
CD13	Goat	6	6	1/200	NA	750	Polyclonal	R&D Systems	Cell	Yes	AF2335	AB_2227288
TNC	Rat	6	6	1/100	NA	647	MTn-12	Thermo Fisher Scientific	Cell	No	MA1-26778	AB_2256026
NeuN	Rabbit	6	6	1/200	NA	555	EPR12763	Abcam	Nucleus	Yes	ab177487	AB_2532109
NF-H	Chick	6	6	1/400	NA	488	Polyclonal	Novus Biologicals	Cell	Yes	NB300-217	AB_350531
CD31	Goat	7	7	1/100	NA	750	Polyclonal	R&D Systems	Cell	Yes	AF806	AB_355617
CC3	Rabbit	7	7	1/100	NA	647	5A1E	Cell Signaling	Nucleus	No	9664S	AB_2070042
GFAP	Chick	7	7	1/400	NA	488	Polyclonal	Abcam	Nucleus	Yes	ab4674	AB_304558
s100b	Rabbit	8	8	1/400	NA	750	EP1576Y	Abcam	Nucleus	Yes	ab52642	AB_882426
S100A8	Goat	8		1/400	NA	647	Polyclonal	R&D Systems	Cell	Yes	AF3059	AB_2184254
Podoplanin	Hamster	8	8	1/50	PE	555	8.1.1	eBioscience	Cell	Yes	12-5381-82	AB_1907439
aSMA	Mouse	8	8	1/100	488	488	1A4	Abcam	Cell	Yes	ab184675	AB_2832195
MPO	Goat	9	8	1/300	NA	750	Polyclonal	R&D Systems	Cell	Yes	AF3667	AB_2250866
NG2	Rabbit	9		1/200	NA	647	Polyclonal	Millipore	Cell	No	AB5320	AB_11213678
Laminin	Rabbit	9		1/50	488	488	Polyclonal	Novus Biologicals	Cell	No	NB300-144AF488	AB_10001146
Periostin	Rabbit	10		1/300	NA	750	Polyclonal	Abcam	Cell	No	ab14041	AB_2299859
RedDot2	NA	10		1/100	647	647	NA	Biotium	Nucleus	No	40061-T	NA
CSPG5	Goat	10		1/200	NA	555	Polyclonal	R&D Systems	Cell	No	AF5665	AB_2087894
Desmin	Rabbit	10		1/100	488	488	Y66	Abcam	Cell	Yes	ab32362	AB_731901
Fibronectin	Rabbit	11		1/200	NA	750	Polyclonal	Abcam	Cell	No	ab2413	AB_2262874
Col IV	Goat	11		1/200	NA	647	Polyclonal	Bio-Rad	Cell	No	134001	AB_2082646
WGA	NA	11		1/200	555	555	NA	Thermo Fisher Scientific	Cell	No	W32466	NA
aTubulin	Chick	11		1/400	488	488	Polyclonal	Abcam	Cell	No	ab89984	NA
Col I	Rabbit	12		1/200	NA	750	Polyclonal	Abcam	Cell	No	ab34710	AB_731684
VE-Cad	Goat	12		1/200	NA	647	Polyclonal	R&D Systems	Cell	Yes	AF1002	AB_2077789
Phalloidin	NA	12		1/400	488	488	NA	Thermo Fisher Scientific	Cell	No	A12379	NA
Ck14	Chick		1	1/500	NA	488	Polyclonal	BioLegend	Cell	Yes	906004	AB_2616962
Ck8	Rabbit		3	1/100	NA	750	EP1628Y	Abcam	Cell	Yes	ab53280	AB_869901
E-cadherin	Rat		4	1/400	NA	750	ECCD-2	Thermo Fisher Scientific	Cell	Yes	13-1900	AB_86571
Epcam	Rabbit		4	1/100	NA	555	Polyclonal	Abcam	Cell	Yes	ab71916	AB_1603782

Supplementary Table 2. HIFI Marker Panels. List of antibodies used in HIFI panel, including the species in which the antibody was raised, clone number, conjugation status, dilution, supplier, RRID, order of imaging, fluorescence channel, and object area used for MFI measurements.

Supplementary Note 1

Validation of antibodies in our manuscript:

- Rat anti-mouse CD3 has been validated for IF by the manufacturer and reported in at least 9 previous publications, including: Nature. 2021 February ; 590(7846): 473–479.
- Rat anti-mouse CD8a has been validated for IF by the manufacturer: "53-6.7 monoclonal antibody specifically binds to the 38 kDa α and 34 kDa α' chains of the CD8 differentiation antigen (Ly-2 or Lyt-2) of all mouse strains tested". It was reported in at least 25 previous publications, including: Science. 2001; 294(5548):1848-1849.
- Rabbit anti-mouse Iba1 has been validated for IHC by the manufacturer: "Fujifilm Wako's Anti Iba1, Rabbit (for immunocytochemistry) (Product Number 019-19741), which allows even microglia processes to be stained by immunohistochemical staining, is used by researchers all over the world as a microglia marker antibody standard." It was reported in at least 4160 previous studies, including: Cell Rep. 2020 Jul 7; 32(1): 107864.
- Chick anti-GFP has been validated for IF by the manufacturer: "Our GFP antibody does cross-react with the many fluorescent proteins that are derived from the jellyfish Aequorea victoria. These are all proteins that differ from the original GFP by just a few point mutations (EGFP, YFP, mVenus, CFP, BFP etc.)." It was reported in at least 3182 previous studies, including: Neuron 111:372-386.e4 (2023).
- Rat anti-mouse CD68 has been validated for IF by the manufacturer: "Rat anti mouse CD68 antibody, clone FA-11, has been used in many mouse models for the identification of CD68 in immunohistochemical assays, using both frozen and paraffin-embedded tissues (Masaki et al. 2003) and (Devey et al. 2009)." It was reported in at least 228 previous studies, including: J Exp Med. 2023 Jan 2;220(1):e20220654.
- Rabbit anti-mouse P2RY12 has been validated for IHC by the manufacturer and reported in at least 6 previous studies, including: Nature Neurosc. 9(12): 1512-1519.
- Rat anti-mouse CD45 has been validated for flow cytometry by the manufacturer and for IHC by the community. According to the manufacturer: "Each lot of this antibody is quality control tested by immunofluorescent staining with flow cytometric analysis." It was reported in at least 165 previous studies, including: P. Natl. Acad. Sci. USA 98:13306.
- Goat anti-mouse CD206 has been validated for IHC by the manufacturer. It was reported in at least 87 previous studies, including: J Neuroinflammation. 2021 Oct 13;18(1):227.
- Rabbit anti-mouse FOXP1 has been validated for IF by the manufacturer and was knockdown-validated by the manufacturer.
- Rat anti-mouse LY6B has been validated for IF by the manufacturer, with demonstrated reactivity in C57BL6 mice: "Rat anti mouse Ly-6B.2, clone 7/4 recognizes the Ly-6B.2 antigen in 129J; AKR; C57BL/6; C57BL/10; C58; DBA/2; NZB; NZW; SJL; MFI". It was reported in at least 97 previous studies, including: Nat Commun. 13 (1): 1521.
- Goat anti-mouse HIF1a has been validated for IHC by the manufacturer, with this statement on specificity: "This antibody does not cross-react with ARNT or the related HIF-2-alpha." It was reported in at least 129 previous studies, including: iScience 25:104823 (2022).
- Chick anti-mouse Vimentin has been validated for IF by the manufacturer. It was reported in at least 99 previous studies, including: Cancer Res 82:2403-2416 (2022).
- Goat anti-mouse Olig2 has been validated for IHC by the manufacturer. It was reported in at least 99 previous studies, including: Nat Commun. 2022 Dec 12;13(1):7671.
- Rat anti-mouse Ki67 has been validated for flow cytometry by the manufacturer, and for IHC by the community. It was reported in at least 23 publications, including: Cell Rep. 2022 Jan 18;38(3):110266.
- Rabbit anti-mouse Sox9 has been validated for IF by the manufacturer. It is the carrier free version of ab185966 which was reported in at least 175 previous studies, including: Nat Neurosci 25:596-606 (2022).
- Rat anti-mouse ERTR7 has been validated for IF by the manufacturer. According to the manufacturer "NB100-64932 recognizes ER-TR7, an antigen that is located in the cytoplasm of reticular fibroblasts and is a component of the extracellular matrix of lymphoid and non-lymphoid organs." It was reported in at least 24 previous studies, including: Immunity. 2023 Aug 8;56(8):1778-1793.e10.
- Goat anti-mouse Sox2 has been validated for IHC by the manufacturer. It was reported in at least 203 previous studies, including: Cell Rep. 2020-04-14;31(2):107504.
- Rabbit anti-mouse PDGFRB has been validated for IF by the manufacturer. It was reported in at least 290 previous studies, including: J Exp Med. 2021 Aug 2;218(8):e20210040.
- Chick anti-mouse Lamin AC has been validated for IF by the manufacturer. The manufacturer reports validation data for IF in human HeLa cells and for Western blot in mouse NIH/3T3 cells.
- Goat anti-mouse CD13 has been validated for IHC by the manufacturer. It was reported in at least 32 previous studies, including: Nature. 2022 Nov;611(7936):585-593.
- Rat anti-mouse Tenascin C has been validated for IHC by the manufacturer. It was reported in at least one previous study: iScience. 2021 Dec 11;25(1):103616.
- Rabbit anti-mouse NeuN has been validated for IF by the manufacturer. It was reported in at least 695 previous studies, including: Neuron. 2023 Jan 18;111(2):190-201.e8.
- Chick anti-mouse NF-H has been validated for IF by the manufacturer. It was reported in at least 7 previous publications, including: J Hand Surg Am. 2015 Oct;40(10):2007-16.
- Goat anti-mouse CD31 has been validated for IHC by the manufacturer. It was reported in at least 234 previous studies, including: Nat Commun. 2023 Aug 16;14(1):4965

Supplementary Note 1 - Continued

Validation of antibodies in our manuscript:

- Rabbit anti-mouse CC3 has been validated for IF by the manufacturer. It was reported in at least 5704 previous studies, including: J Exp Med. 2019 Sep 2;216(9):2184-2201.
- Chick anti-mouse GFAP has been validated for IHC by the manufacturer. It was reported in at least 522 previous studies, including: Nat Commun 13:843 (2022).
- Rabbit anti-mouse S100b has been validated for IF by the manufacturer. It was reported in at least 310 previous studies, including: Nat Neurosci 25:106-115 (2022).
- Goat anti-mouse S100A8 has been validated for Western blot by the manufacturer and for IHC by the research community. It was reported in at least 21 previous studies, including: Am J Pathol, 2014-04-13;184(6):1877-89.
- Hamster anti-mouse podoplanin has been validated for flow cytometry by the manufacturer and for IF by the research community. It was reported in at least 29 previous publications, including: Nat Neurosci. 2018 Oct;21(10):1380-1391.
- Mouse anti-mouse aSMA has been validated for IF by the manufacturer. It was reported in at least 15 previous studies, including: Nat Commun 13:6672 (2022).
- Goat anti-mouse MPO has been validated for IHC by the manufacturer. It was reported in at least 79 previous publications, including: Nat Commun. 2022 Jul 25;13(1):4170.
- Rabbit anti-mouse NG2 has been validated for IHC by the manufacturer. It was reported in at least. According to the manufacturer “AB5320 identifies both the intact proteoglycan and the core protein by Western blot and ELISA. When oligodendrocyte precursor cells (i.e. O-2A progenitor cells) are stained alive, the stain appears as clusters on the cell surface. This antibody does not stain differentiated oligodendrocytes well.” It was reported in at least 787 previous studies, including: Cancer Discov. 2021 Feb;11(2):424-445.
- Rabbit anti-mouse laminin has been validated for IF by the manufacturer. It was reported in at least 117 previous publications, including: Cancer Res. 2009 May 15;69(10):4537-44.
- Rabbit anti-mouse periostin has been validated for IF by the manufacturer. It was reported in at least 193 previous studies, including: Nat Commun 13:4166 (2022).
- Goat anti-mouse CSPG5 has been validated for IF by the manufacturer. It was reported in at least 7 previous studies, including: J Biol Chem. 2000 Jan 7;275(1):337-42.
- Rabbit anti-mouse desmin has been validated for IF by the manufacturer. It was reported in at least 151 previous studies, including: Nat Commun 13:6672 (2022).
- Rabbit anti-mouse fibronectin has been validated for IF by the manufacturer. It was reported in at least 832 previous studies, including: J Clin Invest. 2022 Dec 15;132(24):e159672.
- Goat anti-mouse collagen IV has been validated for IF by the manufacturer. It was reported in at least 6 previous studies, including: J Neuroinflammation. 2019 Jul 27;16(1):157.
- Chick anti-mouse aTubulin has been validated for IF by the manufacturer. It was reported in at least 55 previous studies, including: Mol Cell Neurosci. 2016 Jul:74:58-70.
- Rabbit anti-mouse collagen I has been validated for IHC in human by the manufacturer, and for IF in mice by the research community. It was reported in at least 1659 previous studies, including: J Clin Invest. 2022 Jun 1;132(11):e154092.
- Goat anti-mouse VE-cadherin has been validated for IHC by the manufacturer. It was reported in at least 14 previous studies, including: Blood. 2006 Dec 15;108(13):4018-24.
- Chick anti-mouse Ck14 has been validated for IHC by the manufacturer. It was reported in at least 34 previous studies, including: Immunity. 2024 Jan 9;57(1):124-140.e7.
- Rabbit anti-mouse Ck8 has been validated for IF by the manufacturer. It was reported in at least 105 previous studies, including: Nat Commun 13:7860 (2022).
- Rat anti-mouse E-cadherin has been validated for IF by the manufacturer. It was reported in at least 222 previous publications, including: Nature. 2021 Jan;589(7842):448-455.
- Rabbit anti-mouse Epcam has been validated for IF by the manufacturer. It was reported in at least 145 previous studies, including: Nat Commun 11:3929 (2020).
