# Supplementary material for: Microenvironmental reorganization in brain tumors following radiotherapy and recurrence revealed by hyperplexed immunofluorescence imaging
Source: Nat Commun. 2024 Apr 15;15:3226. doi: 10.1038/s41467-024-47185-9 (PMC11018859; doi:10.1038/s41467-024-47185-9)
Supplement: Supplementary file 2 — Reporting Summary [file 41467_2024_47185_MOESM2_ESM.pdf]

nature portfolio

Corresponding author(s): Johanna Joyce, Spencer S Watson

Last updated by author(s): Mar 1, 2023

Reporting Summary

Nature Portfolio wishes to improve the reproducibility of the work that we publish. This form provides structure for consistency and transparency in reporting. For further information on Nature Portfolio policies, see our Editorial Policies and the Editorial Policy Checklist.

Statistics

For all statistical analyses, confirm that the following items are present in the figure legend, table legend, main text, or Methods section.

n/a	Confirmed	
[ ]	[x]	The exact sample size (n) for each experimental group/condition, given as a discrete number and unit of measurement
[ ]	[x]	A statement on whether measurements were taken from distinct samples or whether the same sample was measured repeatedly
[ ]	[x]	The statistical test(s) used AND whether they are one- or two-sided<br>*Only common tests should be described solely by name; describe more complex techniques in the Methods section.*
[x]	[ ]	A description of all covariates tested
[ ]	[x]	A description of any assumptions or corrections, such as tests of normality and adjustment for multiple comparisons
[ ]	[x]	A full description of the statistical parameters including central tendency (e.g. means) or other basic estimates (e.g. regression coefficient) AND variation (e.g. standard deviation) or associated estimates of uncertainty (e.g. confidence intervals)
[ ]	[x]	For null hypothesis testing, the test statistic (e.g. F, t, r) with confidence intervals, effect sizes, degrees of freedom and P value noted<br>*Give P values as exact values whenever suitable.*
[x]	[ ]	For Bayesian analysis, information on the choice of priors and Markov chain Monte Carlo settings
[x]	[ ]	For hierarchical and complex designs, identification of the appropriate level for tests and full reporting of outcomes
[x]	[ ]	Estimates of effect sizes (e.g. Cohen's d, Pearson's r), indicating how they were calculated

Our web collection on statistics for biologists contains articles on many of the points above.

Software and code

Policy information about availability of computer code

Data collection	Image acquisition and basic post-processing was performed with the commercial software Zeiss Zen package (blue edition, version 3.6.095). Sequential image alignment was performed with the custom open source HIFI Alignment Tool (https://github.com/jhausserlab/HiFImageProcessing). This code is executed in Python version 4.1.2, and all necessary Python packages are detailed on the Github wiki.
Data analysis	HIFI image analysis, machine learning region annotation, deep learning cell segmentation, and all measurements were performed in QuPath version 0.4.4, using the java runtime environment version 16.0.2. Subsequent analysis of image measurements were performed with the commercial statistical software Graph Pad Prism version 9.4.1, and the open source statistical analysis environment R version 4.1.2 using RStudio version 2022.07.02 build 576. Key R packages employed are the following: imcRtools version 1.0.2, spatial Experiment version 1.4.0, singleCellExperiment version 1.16.0, pheatmap version 1.0.12, and igraph version 1.3.5.<br><br>Brief description of analytical pipeline: HIFI images were analyzed in QuPath. For each image multiple pixel classifiers were trained to identify the entire lesion, and structural features of the tissue. Due to the inherent uncertainty of seedless machine learning, these features cannot be precisely reproduced by each user. However, the pertinent settings to achieve comparable results are provided in the Methods section. Following training, each lesion is detected in each image, then the Cell_Detection.groovy script is run in QuPath using the Joyce Lab Best_model_270521 trained model for cell segmentation and cell feature measurement. Next, the Region_Annotation.groovy script is executed to annotate structural features in tumors.<br><br>FlowSOM semi-supervised clustering is performed as described in the Methods section and the data repository to generate cell classifications for each single-cell object with the pipeline in FlowSOM_PDG_BrM.qmd. These classifications are reimported into the QuPath project using

the Cell_labeling_QuPath_v4x.groovy.

Single cell measurements of all images were exported from QuPath for analysis in R. Proximity network analysis was performed with the Proximity_Network_Analysis.qmd R notebook. Subsequent neighborhood analysis was performed with the Neighborhood_Analysis.qmd R notebook.

All code, scripts, deep learning models, and processed data are available at the following Zenodo repository, DOI: 10.5281/zenodo.10778429. Training data and iMAXT StarDist nuclear detection model are available from the following Github repository: https://github.com/TristanWhitmarsh/IMAXT_StarDist_Cellpose.

For manuscripts utilizing custom algorithms or software that are central to the research but not yet described in published literature, software must be made available to editors and reviewers. We strongly encourage code deposition in a community repository (e.g. GitHub). See the Nature Portfolio guidelines for submitting code & software for further information.

Data

Policy information about availability of data

All manuscripts must include a data availability statement. This statement should provide the following information, where applicable:

- Accession codes, unique identifiers, or web links for publicly available datasets
- A description of any restrictions on data availability
- For clinical datasets or third party data, please ensure that the statement adheres to our policy

All raw mage data measurements, statistics, and code (R and Groovy) are available in the following Zenodo repository, DOI: 10.5281/zenodo.10778429. All processed data needed to replicate the analyses are included in the repository. HIFI image data size exceeds online hosting capacities, and so are available upon request to the authors.

Research involving human participants, their data, or biological material

Policy information about studies with human participants or human data. See also policy information about sex, gender (identity/presentation), and sexual orientation and race, ethnicity and racism.

Reporting on sex and gender	NA
Reporting on race, ethnicity, or other socially relevant groupings	NA
Population characteristics	NA
Recruitment	NA
Ethics oversight	NA

Note that full information on the approval of the study protocol must also be provided in the manuscript.

Field-specific reporting

Please select the one below that is the best fit for your research. If you are not sure, read the appropriate sections before making your selection.

☒ Life sciences ☐ Behavioural & social sciences ☐ Ecological, evolutionary & environmental sciences

For a reference copy of the document with all sections, see nature.com/documents/nr-reporting-summary-flat.pdf

Life sciences study design

All studies must disclose on these points even when the disclosure is negative.

Sample size	The size of each experimental group was based on our previous studies and statistical analysis to minimize the number of animals needed to observe the statistically significant changes in tumor growth, response to treatment, and subject survival. (Quail et al.,Science 2016, Croci et al. Science Translational Medicine 2022, Pyonteck et al. Nature Medicine 2013, Bejarano et al. Cancer Cell 2024) We used the statistical program Openepi (www.openepi.com), to assess sample size and mean difference to evaluate the size of each group in view of the expected mean and SEM taken from previous similar experiments. Based on our multiple previous studies cited in the manuscript, we determined the that the group size and degree of sampling was sufficient to represent the heterogeneity inherent in both our murine glioma model, and our murine brain metastasis model.
Data exclusions	3 HIFI images were excluded from the final analysis based on failing the described quality control measures
Replication	Biological replicates (n=3) and technical replicates (n=4) were included in the reported study. The methodology to generate the data using the described workflow has been repeated 50+ times. All replication attempts were successful in the absense of user error.
Randomization	Mice with developed tumors were selected at random from a cohort of age-matched mice with concurrently initiated tumors. These mice were further randomly selected for each of the three treatment arms.

Blinding	Blinding was not relevant to this study as mice were randomly assigned to treatment arms, and knowledge of treatment status during monitoring was required for the health and wellbeing of the experimental subject.

Reporting for specific materials, systems and methods

We require information from authors about some types of materials, experimental systems and methods used in many studies. Here, indicate whether each material, system or method listed is relevant to your study. If you are not sure if a list item applies to your research, read the appropriate section before selecting a response.

Materials & experimental systems

n/a	Involved in the study
☐	☒ Antibodies
☐	☒ Eukaryotic cell lines
☒	☐ Palaeontology and archaeology
☐	☒ Animals and other organisms
☒	☐ Clinical data
☒	☐ Dual use research of concern
☒	☐ Plants

Methods

n/a	Involved in the study
☒	☐ ChIP-seq
☒	☐ Flow cytometry
☐	☒ MRI-based neuroimaging

Antibodies

Antibodies used

CD3   1/50 KT3 Abcam AB33429 AB_726330
CD8a   1/50 53-6.7 BD Pharmingen 557682 AB_396792
Iba1   1/400 Polyclonal Wako 019-19741 AB_839504
GFP   1/500 Polyclonal Abcam AB13970 AB_300798
CD68   1/100 FA-11 Bio-Rad MCA1957 AB_322219
P2RY12   1/200 Polyclonal Anawa AS-55043A NA
CD45   1/30 30-F11 BioLegend 103106 AB_312971
CD206   1/200 Polyclonal R&D Systems AF2535 AB_2063012
FoxP1   1/100 Polyclonal Novus Biologicals NBP1-89410 AB_11023624
Ly6b   1/50 7/4 BioRad MCA771A AB_1102791
HIF1a   1/200 Polyclonal Abcam ab16066 AB_302234
Vimentin   1/300 Polyclonal Abcam ab24525 AB_778824
Olig2   1/100 Polyclonal R&D Systems AF2418 AB_2157554
Ki67   1/50 SolA15 Thermo Fisher Scientific 17-5698-82 AB_2688057
Sox9   1/100 EPR14335-78 Abcam ab225541 AB_3073665
ERTR7   1/400 Polyclonal BioTechne NB100-64932 AB_963381
Sox2   1/200 Polyclonal R&D Systems AF2018 AB_355110
PDGFRB   1/300 Y92 Abcam ab32570 AB_777165
Lamin AC   1/150 Polyclonal Antibodies.com A85443 AB_2748991
CD13   1/200 Polyclonal R&D Systems AF2335 AB_2227288
TNC   1/100 MTn-12 Thermo Fisher Scientific MA1-26778 AB_2256026
NeuN   1/200 EPR12763 Abcam ab177487 AB_2532109
NF-H   1/400 Polyclonal Novus Biologicals NB300-217 AB_350531
CD31   1/100 Polyclonal R&D Systems AF806 AB_355617
CC3   1/100 5A1E Cell Signaling 9664S AB_2070042
GFAP   1/400 Polyclonal Abcam ab4674 AB_304558
s100b   1/400 EP1576Y Abcam ab52642 AB_882426
S100A8   1/400 Polyclonal R&D Systems AF3059 AB_2184254
Podoplanin   1/50 8.1.1 eBioscience 12-5381-82 AB_1907439
aSMA   1/100 1A4 Abcam ab184675 AB_2832195
MPO   1/300 Polyclonal R&D Systems AF3667 AB_2250866
NG2   1/200 Polyclonal Millipore AB5320 AB_11213678
Laminin   1/50 Polyclonal Novus Biologicals NB300-144AF488 AB_10001146
Periostin   1/300 Polyclonal Abcam ab14041 AB_2299859
CSPG5   1/200 Polyclonal R&D Systems AF5665 AB_2087894
Desmin   1/100 Y66 Abcam ab32362 AB_731901
Fibronectin   1/200 Polyclonal Abcam ab2413 AB_2262874
Col IV   1/200 Polyclonal Bio-Rad 134001 AB_2082646
aTubulin   1/400 Polyclonal Abcam ab89984 NA
Col I   1/200 Polyclonal Abcam ab34710 AB_731684
VE-Cad   1/200 Polyclonal R&D Systems AF1002 AB_2077789
Ck14   1/500 Polyclonal BioLegend 906004 AB_2616962
Ck8   1/100 EP1628Y Abcam ab53280 AB_869901

	E-cadherin 1/400 ECCD-2 Thermo Fisher Scientific 13-1900 AB_86571<br>Epcam 1/100 Polyclonal Abcam ab71916 AB_1603782

Validation

Validation of antibodies in our manuscript:
• Rat anti-mouse CD3 has been validated for IF by the manufacturer and reported in at least 9 previous publications, including: Nature. 2021 February ; 590(7846): 473–479.
• Rat anti-mouse CD8a has been validated for IF by the manufacturer: "53-6.7 monoclonal antibody specifically binds to the 38 kDa α and 34 kDa α' chains of the CD8 differentiation antigen (Ly-2 or Lyt-2) of all mouse strains tested". It was reported in at least 25 previous publications, including: Science. 2001; 294(5548):1848-1849.
• Rabbit anti-mouse Iba1 has been validated for IHC by the manufacturer: "Fujifilm Wako's Anti Iba1, Rabbit (for immunocytochemistry) (Product Number 019-19741), which allows even microglia processes to be stained by immunohistochemical staining, is used by researchers all over the world as a microglia marker antibody standard." It was reported in at least 4160 previous studies, including: Cell Rep. 2020 Jul 7; 32(1): 107864.
• Chick anti-GFP has been validated for IF by the manufacturer: "Our GFP antibody does cross-react with the many fluorescent proteins that are derived from the jellyfish Aequorea victoria. These are all proteins that differ from the original GFP by just a few point mutations (EGFP, YFP, mVenus, CFP, BFP etc.)." It was reported in at least 3182 previous studies, including: Neuron 111:372-386.e4 (2023).
• Rat anti-mouse CD68 has been validated for IF by the manufacturer: "Rat anti mouse CD68 antibody, clone FA-11, has been used in many mouse models for the identification of CD68 in immunohistochemical assays, using both frozen and paraffin-embedded tissues (Masaki et al. 2003) and (Devey et al. 2009)." It was reported in at least 228 previous studies, including: J Exp Med. 2023 Jan 2;220 (1):e20220654.
• Rabbit anti-mouse P2RY12 has been validated for IHC by the manufacturer and reported in at least 6 previous studies, including: Nature Neurosc. 9(12): 1512-1519.
• Rat anti-mouse CD45 has been validated for flow cytometry by the manufacturer and for IHC by the community. According to the manufacturer: "Each lot of this antibody is quality control tested by immunofluorescent staining with flow cytometric analysis." It was reported in at least 165 previous studies, including: P. Natl. Acad. Sci. USA 98:13306.
• Goat anti-mouse CD206 has been validated for IHC by the manufacturer. It was reported in at least 87 previous studies, including: J Neuroinflammation. 2021 Oct 13;18(1):227.
• Rabbit anti-mouse FOXP1 has been validated for IF by the manufacturer and was knockdown-validated by the manufacturer.
• Rat anti-mouse LY6B has been validated for IF by the manufacturer, with demonstrated reactivity in C57BL6 mice: "Rat anti mouse Ly-6B.2, clone 7/4 recognizes the Ly-6B.2 antigen in 129J; AKR; C57BL/6; C57BL/10; C58; DBA/2; NZB; NZW; SJL; MFI". It was reported in at least 97 previous studies, including: Nat Commun. 13 (1): 1521.
• Goat anti-mouse HIF1a has been validated for IHC by the manufacturer, with this statement on specificity: "This antibody does not cross-react with ARNT or the related HIF-2-alpha." It was reported in at least 129 previous studies, including: iScience 25:104823 (2022).
• Chick anti-mouse Vimentin has been validated for IF by the manufacturer. It was reported in at least 99 previous studies, including: Cancer Res 82:2403-2416 (2022).
• Goat anti-mouse Olig2 has been validated for IHC by the manufacturer. It was reported in at least 99 previous studies, including: Nat Commun. 2022 Dec 12;13(1):7671.
• Rat anti-mouse Ki67 has been validated for flow cytometry by the manufacturer, and for IHC by the community. It was reported in at least 23 publications, including: Cell Rep. 2022 Jan 18;38(3):110266.
• Rabbit anti-mouse Sox9 has been validated for IF by the manufacturer. It is the carrier free version of ab185966 which was reported in at least 175 previous studies, including: Nat Neurosci 25:596-606 (2022).
• Rat anti-mouse ERTR7 has been validated for IF by the manufacturer. According to the manufacturer "NB100-64932 recognizes ER-TR7, an antigen that is located in the cytoplasm of reticular fibroblasts and is a component of the extracellular matrix of lymphoid and non-lymphoid organs." It was reported in at least 24 previous studies, including: Immunity. 2023 Aug 8;56(8):1778-1793.e10.
• Goat anti-mouse Sox2 has been validated for IHC by the manufacturer. It was reported in at least 203 previous studies, including: Cell Rep. 2020-04-14;31(2):107504.
• Rabbit anti-mouse PDGFRB has been validated for IF by the manufacturer. It was reported in at least 290 previous studies, including: J Exp Med. 2021 Aug 2;218(8):e20210040.
• Chick anti-mouse Lamin AC has been validated for IF by the manufacturer. The manufacturer reports validation data for IF in human HeLa cells and for Western blot in mouse NIH/3T3 cells.
• Goat anti-mouse CD13 has been validated for IHC by the manufacturer. It was reported in at least 32 previous studies, including: Nature. 2022 Nov;611(7936):585-593.
• Rat anti-mouse Tenascin C has been validated for IHC by the manufacturer. It was reported in at least one previous study: iScience. 2021 Dec 11;25(1):103616.
• Rabbit anti-mouse NeuN has been validated for IF by the manufacturer. It was reported in at least 695 previous studies, including: Neuron. 2023 Jan 18;111(2):190-201.e8.
• Chick anti-mouse NF-H has been validated for IF by the manufacturer. It was reported in at least 7 previous publications, including: J Hand Surg Am. 2015 Oct;40(10):2007-16.
• Goat anti-mouse CD31 has been validated for IHC by the manufacturer. It was reported in at least 234 previous studies, including: Nat Commun. 2023 Aug 16;14(1):4965.
• Rabbit anti-mouse CC3 has been validated for IF by the manufacturer. It was reported in at least 5704 previous studies, including: J Exp Med. 2019 Sep 2;216(9):2184-2201.
• Chick anti-mouse GFAP has been validated for IHC by the manufacturer. It was reported in at least 522 previous studies, including: Nat Commun 13:843 (2022).
• Rabbit anti-mouse S100b has been validated for IF by the manufacturer. It was reported in at least 310 previous studies, including: Nat Neurosci 25:106-115 (2022).
• Goat anti-mouse S100A8 has been validated for Western blot by the manufacturer and for IHC by the research community. It was reported in at least 21 previous studies, including: Am J Pathol, 2014-04-13;184(6):1877-89.
• Hamster anti-mouse podoplanin has been validated for flow cytometry by the manufacturer and for IF by the research community. It was reported in at least 29 previous publications, including: Nat Neurosci. 2018 Oct;21(10):1380-1391.

• Mouse anti-mouse aSMA has been validated for IF by the manufacturer. It was reported in at least 15 previous studies, including: Nat Commun 13:6672 (2022).
• Goat anti-mouse MPO has been validated for IHC by the manufacturer. It was reported in at least 79 previous publications, including: Nat Commun. 2022 Jul 25;13(1):4170.
• Rabbit anti-mouse NG2 has been validated for IHC by the manufacturer. It was reported in at least. According to the manufacturer "AB5320 identifies both the intact proteoglycan and the core protein by Western blot and ELISA. When oligodendrocyte precursor cells (i.e. O-2A progenitor cells) are stained alive, the stain appears as clusters on the cell surface. This antibody does not stain differentiated oligodendrocytes well." It was reported in at least 787 previous studies, including: Cancer Discov. 2021 Feb;11 (2):424-445.
• Rabbit anti-mouse laminin has been validated for IF by the manufacturer. It was reported in at least 117 previous publications, including: Cancer Res. 2009 May 15;69(10):4537-44.
• Rabbit anti-mouse periostin has been validated for IF by the manufacturer. It was reported in at least 193 previous studies, including: Nat Commun 13:4166 (2022).
• Goat anti-mouse CSPG5 has been validated for IF by the manufacturer. It was reported in at least 7 previous studies, including: J Biol Chem. 2000 Jan 7;275(1):337-42.
• Rabbit anti-mouse desmin has been validated for IF by the manufacturer. It was reported in at least 151 previous studies, including: Nat Commun 13:6672 (2022).
• Rabbit anti-mouse fibronectin has been validated for IF by the manufacturer. It was reported in at least 832 previous studies, including: J Clin Invest. 2022 Dec 15;132(24):e159672.
• Goat anti-mouse collagen IV has been validated for IF by the manufacturer. It was reported in at least 6 previous studies, including: J Neuroinflammation. 2019 Jul 27;16(1):157.
• Chick anti-mouse aTubulin has been validated for IF by the manufacturer. It was reported in at least 55 previous studies, including: Mol Cell Neurosci. 2016 Jul:74:58-70.
• Rabbit anti-mouse collagen I has been validated for IHC in human by the manufacturer, and for IF in mice by the research community. It was reported in at least 1659 previous studies, including: J Clin Invest. 2022 Jun 1;132(11):e154092.
• Goat anti-mouse VE-cadherin has been validated for IHC by the manufacturer. It was reported in at least 14 previous studies, including: Blood. 2006 Dec 15;108(13):4018-24.
• Chick anti-mouse Ck14 has been validated for IHC by the manufacturer. It was reported in at least 34 previous studies, including: Immunity. 2024 Jan 9;57(1):124-140.e7.
• Rabbit anti-mouse Ck8 has been validated for IF by the manufacturer. It was reported in at least 105 previous studies, including: Nat Commun 13:7860 (2022).
• Rat anti-mouse E-cadherin has been validated for IF by the manufacturer. It was reported in at least 222 previous publications, including: Nature. 2021 Jan;589(7842):448-455.
• Rabbit anti-mouse Epcam has been validated for IF by the manufacturer. It was reported in at least 145 previous studies, including: Nat Commun 11:3929 (2020).

Eukaryotic cell lines

Policy information about cell lines and Sex and Gender in Research

Cell line source(s)	the DF1-PDG-GFP chicken fibroblast cell line was developed in house. The PyMT-BrM3 breast cell line was derived from the murine parental 99LN cell line, which was isolated from a metastatic lymph node lesion that arose in the MMTV-PyMT (murine mammary tumor virus; polyoma middle T antigen) breast cancer model (C57BL/6J background). This cell line was sequentially selected three times in vivo for brain-homing capacity, resulting in the PyMT-BrM3 variant used herein.
Authentication	Cell line authentication consisted of routine microscopic morhpology checks, growth curve assessment, and mycoplasma testing for matched passage numbers.
Mycoplasma contamination	All cell lines were routinely tested for mycoplasma infection, and at no time was infection detected in any cell line.
Commonly misidentified lines (See ICLAC register)	NA

Animals and other research organisms

Policy information about studies involving animals; ARRIVE guidelines recommended for reporting animal research, and Sex and Gender in Research

Laboratory animals	Gliomas were initiated in murine GEMM strain C57BL/6-Nestin-Tv-a;Ink4a/Arf-/- (Tg(NES-TVA); Cdkn2atm1Rdp) mice at 5 weeks of age. Breast-to-brain metastases were initiated in C57BL/6 mice at 5 weeks of age. Mice were housed in the Agora In Vivo Center (AIVC) animal facility in individually ventilated cages, under a 12h light/dark schedule at 22°C and in the presence of 2-4 cage mates. Standard autoclaved lab diet and water were provided ad libitum.
Wild animals	No wild animals used in the study.
Reporting on sex	No sex-based differences were observed in the glioma model, and the cohort had random age-matched cohorts of both sexes. The breast-to-brain metastasis model inlcuded only female mice due to the tumor cell line being female in origin.
Field-collected samples	No field-collected samples were used in this study.
Ethics oversight	All animal studies were approved by the Institutional Animal Care and Use Committees of the University of Lausanne and Canton

Vaud, Switzerland (License numbers: VD3804 and VD3688).

Note that full information on the approval of the study protocol must also be provided in the manuscript.

Plants

Seed stocks	NA
Novel plant genotypes	NA
Authentication	NA

Magnetic resonance imaging

Experimental design

Design type	Monitoring and lesion volume measurement only
Design specifications	NA
Behavioral performance measures	NA

Acquisition

Imaging type(s)	Structural
Field strength	3 tesla
Sequence & imaging parameters	T2 TurboRARE, no contrast, slice thickness 0.8 mm, slice space 0.9 mm, columns/rows mm, flip angle = 180
Area of acquisition	Whole-brain

Diffusion MRI ☐ Used ☒ Not used

Preprocessing

Preprocessing software	Bruker Para Vision 360 version 3.4
Normalization	NA
Normalization template	NA
Noise and artifact removal	NA
Volume censoring	NA

Statistical modeling & inference

Model type and settings	NA
Effect(s) tested	NA

Specify type of analysis: ☒ Whole brain ☐ ROI-based ☐ Both

Statistic type for inference (See Eklund et al. 2016)	NA
Correction	NA

Models & analysis

n/a	Involved in the study
☒	☐ Functional and/or effective connectivity
☒	☐ Graph analysis
☒	☐ Multivariate modeling or predictive analysis
