# Supplementary material for: Microenvironmental reorganization in brain tumors following radiotherapy and recurrence revealed by hyperplexed immunofluorescence imaging
Source: Nat Commun. 2024 Apr 15;15:3226. doi: 10.1038/s41467-024-47185-9 (PMC11018859; doi:10.1038/s41467-024-47185-9)
Supplement: Supplementary file 3 — Peer Review File [file 41467_2024_47185_MOESM3_ESM.pdf]

nature portfolio

Peer Review File

Microenvironmental Reorganization in Brain Tumors Following Radiotherapy and Recurrence Revealed by Hyperplexed Immunofluorescence Imaging

Open Access This file is licensed under a Creative Commons Attribution 4.0 International License, which permits use, sharing, adaptation, distribution and reproduction in any medium or format, as long as you give appropriate credit to the original author(s) and the source, provide a link to the Creative Commons license, and indicate if changes were made. In the cases where the authors are anonymous, such as is the case for the reports of anonymous peer reviewers, author attribution should be to 'Anonymous Referee' followed by a clear attribution to the source work. The images or other third party material in this file are included in the article's Creative Commons license, unless indicated otherwise in a credit line to the material. If material is not included in the article's Creative Commons license and your intended use is not permitted by statutory regulation or exceeds the permitted use, you will need to obtain permission directly from the copyright holder. To view a copy of this license, visit http://creativecommons.org/licenses/by/4.0/.

Reviewers' Comments:

Reviewer #1:
Remarks to the Author:
In this interesting manuscript, Watson et al. develop a new protocol for multiplexed immunohistochemistry intended to overcome some of the limitations of current state of the art methods. Their method, as applied in the present study, allows for analysis of 45 independent marker proteins in single tissue sections by cycles of serial stainings and gentle antibody removal. The authors combine this serial staining approach with free image analysis software for cell segmentation and further analyses. They apply this new workflow to the analysis of the glioblastoma (GBM) microenvironment before, during, and after radiotherapy, using a genetically engineered mouse model of GBM, and identify potential differences between primary and recurrent GBM in terms of cellular composition and heterotypic cell-cell interactions. The study is timely considering the increased interest in spatial analysis of solid tumors, as well as in light of the increasing appreciation of therapy-induced remodeling of the tumor microenvironment and its consequences for tumor biology.

From a technical point of view, the authors present impressive results that convincingly demonstrate the feasibility of their approach. While the protocols developed appear to be rather laborious and time consuming compared to some alternative methods, they also have several advantages, especially when cost or availability of materials are limiting factors. The study has several challenges when it comes to the application of the method to the evolution of the GBM microenvironment during radiotherapy, some of which could be addressed with additional experimental data as detailed in the specific comments below.

Limitations of the study include:

1. It would be helpful to have more detail regarding the validation of antibodies and the staining protocols. With regards to the sequencing of panels, how do the authors control that epitopes are preserved after multiple staining cycles – were antibodies used in late cycles tested and compared on fresh tissue? Furthermore, it is unclear how the authors confirm that primary antibodies are indeed removed between staining cycles.

2. Considering the study’s focus on the tumor microenvironment and its response to radiotherapy, it is unfortunate that the mouse model used is on an Ink4a/Arf-/- background. In human GBM, immune cells and other cells of the microenvironment would not be expected to carry the same tumor suppressor gene loss-of-function as the tumor cells. It is likely that the Ink4a/Arf-/- background affects the survival and phenotype of stromal cells in response to radiotherapy in the mouse model, which could have implications for the cellular composition during and after treatment. The conclusions of the study and their implications for human GBM would be strengthened by the analysis of tissue from an additional model in which stromal cells do not carry oncogenic genetic aberrations.

3. The number of tumors analyzed for each condition (n=3) seems low in relation to the considerable intertumor heterogeneity described by the authors, especially in the recurrent group. It is possible that the authors would find more pronounced microenvironmental reorganization if more samples were included in the analyses. Even the authors’ modest conclusion that the recurrent tumors are heterogeneous would require more tumors in order to feel confident about.

4. The authors describe one rebound tumor as having a large population of tumor cells that lost GFP expression, and further state that their methodology was able to reliably identify heterogeneous populations such as tumor cells even if they had lost GFP expression. These conclusions are presumably based on the similar marker expression between this population of GFP-negative cells, and other GFP-positive tumor cell populations. This appears somewhat problematic, as the presence of the GFP marker is the defining feature of tumor cells in the model, at least within the context of the 45 markers studied. Without an additional defining and tumor-specific marker (e.g. other tags on PDGFB), it seems difficult to exclude the possibility of stromal cells presenting with a phenotype that closely resembles tumor cells. Indeed, recruitment of OPC-like cells into murine GBM has been previously described, and within the microenvironment of a

PDGFB-driven tumor it is likely that such cells would take on a similar phenotype to the bona fide tumor cells.

Minor issues

5. Fig 4b segmentation data look very impressive. It would be interesting to see these types of data on a larger tumor area, and including the stainings on which the cell type determinations were made in addition to the DAPI.

6. The results section on pages 4-5 contains several passages that read more like introduction or discussion than results.

Reviewer #2:
Remarks to the Author:
Summary: The authors have presented a multiplexed imaging technology that can image upto 45 markers in a tissue sample. They have demonstrated the technology on samples of a glioblastoma patient before and after radiotherapy. Glioblastoma is an aggressive type of cancer with very poor prognosis. Therefore, new technologies to diagnose, understand and treat glioblastoma are acutely needed. Due to high levels of tumor heterogeneity and multitude of cell types present in the glioblastoma microenvironment, new multiplexed imaging technologies have potential to unravel leading indicators of disease and recurrence. Authors have presented a new technique for multiplexed immunofluorescence by utilizing a gentler thiol-based elution buffer for stripping antibodies. They have also utilized computational techniques such as deep-learning and machine learning for automated tasks such as segmentation and feature extraction. Even though this adds to the capabilities of the field for multiplexed disease evaluation, the extent of novelty compared to existing approaches is not very clear and the following major comments need to be addressed.

Comments to the author:
1. Authors should modify the abstract to include some of their findings. Authors claim "These integrated analyses revealed new insights". What are those insights? Please include briefly in the abstract.
2. Authors state in the introduction-"Highly multiplexed imaging approaches require repeated rounds of staining, imaging, and stripping or quenching of markers which can damage tissue samples and the protein epitopes targeted by antibodies." However, authors are also conducting the similar repeated rounds of staining, imaging, and stripping. Even though, later in the results, it is mentioned that the novelty is primarily coming from the chemistry being used in stripping, this aspect is not clear in the introduction and the claim seems misleading. Please clearly mention in the introduction how the authors' method is superior to other multiplexed imaging in terms of time, signal to noise ratio and cost of imaging?
3. Authors mention: "Multiple days are required to complete a full hyperplexed panel. However, as most modern slide-scanning fluorescence microscopes have capacities for 80-100 slides, the ability to scale up to high-throughput offsets the total time required for an experiment when compared to other highly multiplexed approaches." However, comparing with other multiplexed IF approaches like CODEX, the timescale of staining and imaging still seem to be very slow as compared to existing MIF approaches.
4. The Authors mention that the HIFI alignment tool is only limited by the memory (RAM) of the workstation. Since they are dealing with whole slide images, the RAM requirement would be very high. Please specify if the entire image is being loaded at once, if yes what are the specifications of the used workstation. This might significantly reduce the accessibility of this tool especially for large tissue sections.
5. Fig 3d- The authors selected areas of interest, however they initially discuss how selecting regions of interest can result in a selection bias and under sampling. Please clarify how their approach is different and unaffected by these limitations?
6. Fig 3e- "three biological replicates of rebound tumors demonstrated extensive intertumoral heterogeneity". This is major biological insight authors draw from their results. Please explain this in more by detailing different features in the image and clearly marking those features in the

image. What global insights can one draw from these differences that are useful for diagnostic purposes?
7. Fig 4b- The authors should provide the accuracy achieved by their cell segmentation model trained using transfer learning
8. Authors should explain the biological insights gained from Leiden clustering. It seems like authors have described the methodology of Leiden clustering, but did not explain what they learned from it?
9. Was there stage drift during multiple cycles of probe hybridization, imaging and washing in workflow? How did the authors detect and correct for it?
10. Was there any leaking of fluorescent light from one channel to another? What controls were done to ensure no spectral mixing?
11. Authors should show controls for following:
o Minimization of autofluorescence.
o Effectiveness of washing of probes.
o Minimal off target effects.

Reviewer #3:
Remarks to the Author:
This paper describes an innovative, practical, achievable-for-most-labs, multiplexed imaging approach that enables characterisation of the microenvironment in the spatial dimension. The innovation lies in its ease of use, its compatibility with routine laboratory equipment and reagents, and the development and implementation of software solutions that the authors have vetted in a unique preclinical mouse model of glioma. This set of tools will enable new discovery science in solid cancer biology, in both clinical and preclinical settings. Tools like this, with a low barrier to entry, are much needed to expand the scope of research in tumour-microenvironment interactions, specially the approach the authors have taken, which analyses the TME in an experimentally manipulable model-- crucial to identify features that change with treatment or other interventions. Techniques like imaging mass cytometry and CODEX/Akoya require expensive equipment and reagents, or take a lot longer for data acquisition and analysis. Other spatial technologies like Visium have high depth of coverage, but perhaps that's overkill/not necessary for simply calling and phenotyping cell types accurately. This approach strikes the right balance and will be of great interest to many, perhaps not just to cancer biologists, but to developmental biologists and generally anybody interested in assaying the spatial context using a manageable 45-plex panel of commercially available antibodies.

Having said that, can the authors comment on whether:

1) They could test this system on one additional model, perhaps not to the same level of detail as they have done the PDGfp model. Either another GEMM harbouring different mutations, or perhaps a metastatic brain tumour model. To evaluate whether differences between tumour genotypes, or between primary vs metastatic tumours, can be resolved using this system.

2) They could test the compatibility of this system for evaluating genotype:phenotype clonal relationships in highly intratumourally heterogeneous cancers, using a barcoding strategy delivered with the virus in vivo.

3) Whether experimental therapies that impact the TME (CSF1Ris) or the immune axis (CAR-Ts), change cellular neighbourhood associations.

4) Functional implications of the very interesting switch from CN13 (MDM) to CN6 (MG) in recurrent tumours.

These suggestions could well be beyond the scope of this manuscript, but some small attempt to provide a functional or mechanistic insight using this technique should be made, as it would

greatly add to the impact this paper can have.

RESPONSE TO REVIEWER COMMENTS - NCOMMS-23-04329

We would first like to thank all the peer reviewers for their thoughtful evaluation and constructive suggestions. We strongly believe that addressing their critical feedback has resulted in a further improved manuscript. We have addressed each of the comments below, implemented the reviewers' suggestions, and greatly expanded the dataset to include more biological diversity and a completely new brain tumor model with its own fully validated antibody marker panel. Specifically, we generated samples of a breast-to-brain metastasis model (BrM) treated with radiotherapy for the purpose of both technical validation and the biological comparison of response to radiation therapy in primary and metastatic brain tumor samples from preclinical models.

Our comparison of glioblastoma (GBM) to BrM samples following radiotherapy yielded interesting results that refocused our analysis on investigating their differential responses to treatment. While we observed the same interesting features in GBM as we had focused on in the initial manuscript, we believe that including these new results will further enhance interest for the readers of *Nature Communications* and the broader scientific community. While spatial-omic analysis at this large scale generated many interesting findings, as it did in our initial submission, the reviewer feedback aided us in focusing on the most biologically relevant responses to cancer treatment. Consequently, we have now restructured the text, main figures, and supplemental figures to include these new data. We have also uploaded a version of the revised manuscript with tracking changes visible if the editorial team wish to evaluate the changes.

We believe that the inclusion of this new large dataset, and all the additional improvements in response to reviewer suggestions have further strengthened the manuscript, and we hope you will find it suitable for publication in *Nature Communications*.

Reviewer #1 - Glioblastoma, radiotherapy, TME - (Remarks to the Author):

In this interesting manuscript, Watson et al. develop a new protocol for multiplexed immunohistochemistry intended to overcome some of the limitations of current state of the art methods. Their method, as applied in the present study, allows for analysis of 45 independent marker proteins in single tissue sections by cycles of serial stainings and gentle antibody removal. The authors combine this serial staining approach with free image analysis software for cell segmentation and further analyses. They apply this new workflow to the analysis of the glioblastoma (GBM) microenvironment before, during, and after radiotherapy, using a genetically engineered mouse model of GBM, and identify potential differences between primary and recurrent GBM in terms of cellular composition and heterotypic cell-cell interactions. The study is timely considering the increased interest in spatial analysis of solid tumors, as well as in light of the increasing appreciation of therapy-induced remodeling of the tumor microenvironment and its consequences for tumor biology.

From a technical point of view, the authors present impressive results that convincingly demonstrate the feasibility of their approach. While the protocols developed appear to be rather laborious and time consuming compared to some alternative methods, they also have several advantages, especially when cost or availability of materials are limiting factors. The study has several challenges when it comes to the application of the method to the evolution of the GBM microenvironment during radiotherapy, some of which could be addressed with additional experimental data as detailed in the specific comments below.

We greatly appreciate the reviewer's comments and thoughtful critique. We also agree that the protocols as presented may appear time-consuming and laborious; however, much of this is due to the large scale of the current study. Implementing this approach with lower plex and fewer samples is not an appreciably

different workload than traditional immunostaining workflows. We have thus updated several sections of the manuscript to more clearly demonstrate the scalability of the method. We see this as a benefit to the approach, as it represents a strategy to massively multiply existing imaging protocols, and scale them to high-throughput.

We are grateful to the reviewer for their generally positive assessment of the manuscript, and for their valuable suggestions. We believe these suggestions have resulted in a further improved manuscript.

Limitations of the study include:

1. It would be helpful to have more detail regarding the validation of antibodies and the staining protocols. With regards to the sequencing of panels, how do the authors control that epitopes are preserved after multiple staining cycles – were antibodies used in late cycles tested and compared on fresh tissue? Furthermore, it is unclear how the authors confirm that primary antibodies are indeed removed between staining cycles.

To address this comment, we have included additional details in the revised manuscript (lines 211-223) and performed a new control experiment (Supplemental Fig. 1b). In this experiment, we stained multiple tissue sections (treated and untreated) with a range of primary and secondary antibodies. We then imaged and measured mean fluorescence intensity (MFI) for every single-cell object positive for the cell marker. Following this, we eluted the antibodies, restained with only the secondary antibody, and imaged and measured MFI for the same single-cell objects. This was repeated three times to demonstrate elution efficiency for each round, and also to demonstrate the selection criteria by which we determined the specific round in which each marker should be used.

2. Considering the study's focus on the tumor microenvironment and its response to radiotherapy, it is unfortunate that the mouse model used is on an Ink4a/Arf-/- background. In human GBM, immune cells and other cells of the microenvironment would not be expected to carry the same tumor suppressor gene loss-of-function as the tumor cells. It is likely that the Ink4a/Arf-/- background affects the survival and phenotype of stromal cells in response to radiotherapy in the mouse model, which could have implications for the cellular composition during and after treatment. The conclusions of the study and their implications for human GBM would be strengthened by the analysis of tissue from an additional model in which stromal cells do not carry oncogenic genetic aberrations.

To date, neither our lab or other labs employing the RCAS glioma model with constitutive Inkfa/Arf-/- deletion have reported any effect on stromal cells in the tumor microenvironment, or decreased survival following radiotherapy because of being in the Ink4a/Arf-/- background. However, the reviewer is correct that we cannot completely rule out this possibility with a single model. Thus, in addressing this and another reviewer's suggestion for a comparative tumor type, we have added a breast-to-brain (BrM) metastasis tumor model to the study (PyMT-derived BrM3, generated in our lab). To specifically address this point, we chose a syngeneic mouse model in a completely wildtype background (C57Bl6). We orthotopically injected the PyMT-BrM3 cells in the brain, at the same precise cranial coordinates as the glioblastoma model, and treated tumors in both models at equivalent volumes. We analyzed untreated, 7 days post-radiotherapy, and rebound/regrowth samples (n=3 for each) using a subset of the full antibody panel, and tailored to the BrM tumor microenvironment (Supplemental Figure 1). The results can be found in Figures 2-6, and Supplemental Figures 5 and 7. While we did find evident differences between the two models in their response to radiotherapy, we did not observe decreased survival of stromal populations in the glioblastoma model. On the contrary, we observed increases in certain stromal populations following radiation in glioblastoma models, but not in the wildtype BrM model (Figure 5c).

3. The number of tumors analyzed for each condition (n=3) seems low in relation to the considerable intertumor heterogeneity described by the authors, especially in the recurrent group. It is possible that the authors would find more pronounced microenvironmental reorganization if more samples were included in the analyses. Even the authors' modest conclusion that the recurrent tumors are heterogeneous would require more tumors in order to feel confident about.

We agree that this is a valid concern. While our initial dataset was extremely large in terms of individual single-cell objects, a sample set of n=3 can be considered low in terms of biological variability. To further address tumor heterogeneity, we have expanded the glioblastoma sample set to n=5 biological replicates for each treatment condition (Supplemental Figure 3). We additionally replaced one of the previous tumor rebound samples that had lost GFP expression. This sample was comparable in terms of biology to the other rebound lesions, but was determined to be a technical outlier due to the loss of the fluorescent tracker. This new dataset includes the same range of technical replicates per mouse, resulting in the largest single-cell spatial proteomic dataset we have encountered.

The immense scale of this new dataset forced other changes and optimizations to our analytical pipeline, which resulted in an even more robust methodology as presented in this revision. Our initial Leiden clustering method employing PhenoGraph struggled with datasets of almost 7 million cells, both in terms of time required (more than week per optimization) and in terms of accuracy. We also required a method that could account for potential batch effects between the initial imaging (original submission) and the new imaging of the new samples added - so that both sets could be analyzed together. We decided to employ an optimized semi-supervised method based around FlowSOM that was somewhat more labor-intensive but resulted in greatly improved accuracy and sensitivity (Supplemental Figure 4). This allowed us to further stratify several cell types into multiple phenotypes (Supplemental Figure 5) that had distinct spatial patterning (Supplemental Figure 6). The resulting augmentation in cell type stratification led to greatly increased depth for downstream spatial analysis.

4. The authors describe one rebound tumor as having a large population of tumor cells that lost GFP expression, and further state that their methodology was able to reliably identify heterogeneous populations such as tumor cells even if they had lost GFP expression. These conclusions are presumably based on the similar marker expression between this population of GFP-negative cells, and other GFP-positive tumor cell populations. This appears somewhat problematic, as the presence of the GFP marker is the defining feature of tumor cells in the model, at least within the context of the 45 markers studied. Without an additional defining and tumor-specific marker (e.g. other tags on PDGFB), it seems difficult to exclude the possibility of stromal cells presenting with a phenotype that closely resembles tumor cells. Indeed, recruitment of OPC-like cells into murine GBM has been previously described, and within the microenvironment of a PDGFB-driven tumor it is likely that such cells would take on a similar phenotype to the bona fide tumor cells.

We fully agree with this reviewer's concern about the loss of GFP expression in one of the rebound samples. While the inclusion of multiple other tumor-specific markers had allowed us to still identify these cells as tumor cells without GFP expression, the loss of this critical marker complicated downstream analysis, and meant that we could not rule out that these cells were recruited by the microenvironment (as reported by Dr. Eric Holland's lab some years ago; PMID: 21754979).

As mentioned in response to the previous point, when we expanded the biological replicates for the glioblastoma samples, we replaced this technical outlier with a new biological replicate. The reviewer also makes an excellent point about the ability of the tumor to recruit and transform OPC-like cells, which we have also observed. Due to the improvements in cell classification during the revision, we can now show that GFP-negative cells with an OPC-like phenotype demonstrate sufficiently high levels of

proliferation to identify them as transformed neoplastic cells (Supplemental Figure 5d, 6a, and lines 333-336).

Minor issues

5. Fig 4b segmentation data look very impressive. It would be interesting to see these types of data on a larger tumor area, and including the stainings on which the cell type determinations were made in addition to the DAPI.

Based on this suggestion we have expanded the section relating to cell segmentation to include a larger representative example region (Figure 4b), comparisons to alternative approaches, and statistics for model accuracy. In addition, we have collaborated with a consortium of imaging and machine learning experts to generate a new deep-learning cell segmentation model based on a large-scale initiative to create manually annotated ground-truth training data from multiple murine tumors and healthy organ tissues. Both the training data and segmentation model will be publicly released as a community resource if the manuscript is accepted for publication.

6. The results section on pages 4-5 contains several passages that read more like introduction or discussion than results.

These sections have either been moved to the Introduction, or removed from the manuscript.

Reviewer #2 - Digital & molecular pathology, TIME interactions - (Remarks to the Author):

Summary: The authors have presented a multiplexed imaging technology that can image upto 45 markers in a tissue sample. They have demonstrated the technology on samples of a glioblastoma patient before and after radiotherapy. Glioblastoma is an aggressive type of cancer with very poor prognosis. Therefore, new technologies to diagnose, understand and treat glioblastoma are acutely needed. Due to high levels of tumor heterogeneity and multitude of cell types present in the glioblastoma microenvironment, new multiplexed imaging technologies have potential to unravel leading indicators of disease and recurrence. Authors have presented a new technique for multiplexed immunofluorescence by utilizing a gentler thiol-based elution buffer for stripping antibodies. They have also utilized computational techniques such as deep-learning and machine learning for automated tasks such as segmentation and feature extraction. Even though this adds to the capabilities of the field for multiplexed disease evaluation, the extent of novelty compared to existing approaches is not very clear and the following major comments need to be addressed.

We appreciate the reviewer’s feedback, and fully understand their concerns about the novelty of the approach due to a variety of multiplexed imaging approaches currently available. We have updated both the Introduction and the Discussion to better clarify how the HIFI workflow differs appreciably from existing methodologies and improves other spatial analytical approaches. To briefly summarize, HIFI is unique in that it alone sits in the intersection of the Venn diagram that includes concerns about high-plex, high-magnification, low-cost, high-throughput, non-destructive, easy adoption, whole-slide, and compatibility with extremely fragile tissue samples.

While existing approaches can address several of these concerns, there are currently no other techniques that meet all criteria to produce extremely high quality spatial proteomic data. tCyCIF would be the most comparable approach, but the harsh reagents required to inactivate fluorophores is extremely damaging, especially to lightly-fixed cryo-sectioned tissue. HIFI achieves uncompromised data quality by taking a more time-intensive approach that has been robustly optimized. But as noted by another

reviewer, it makes up for the time investment by the ability to scale to high throughput. This means that this approach can produce more single-cell data in the period of one week than any automated approach, while still being compatible with highly challenging tissue samples.

We thank the reviewer for their suggestions, which we believe have greatly improved the manuscript. Below we will address each of the reviewer's helpful comments.

Comments to the author:
1. Authors should modify the abstract to include some of their findings. Authors claim "These integrated analyses revealed new insights". What are those insights? Please include briefly in the abstract.

To address suggestions from other reviewers, we have refocused our analysis to now highlight a potential survival-promoting spatial niche in gliomas following radiation treatment and compared this reorganizational response to a new sample set of preclinical breast-to-brain metastasis (BrM) treated with radiotherapy. We note the interesting observation that BrM tumors do not show a spatial reorganizational response to radiation, but still receive equivalent survival benefit from treatment, indicating a highly divergent survival mechanism between these tumor types. We believe that focusing on this biological response will both address other reviewer's suggestions, and be of enhanced interest to the research community than our initial more narrow focus. We have modified the abstract to more specifically address these new findings.

2. Authors state in the introduction-"Highly multiplexed imaging approaches require repeated rounds of staining, imaging, and stripping or quenching of markers which can damage tissue samples and the protein epitopes targeted by antibodies." However, authors are also conducting the similar repeated rounds of staining, imaging, and stripping. Even though, later in the results, it is mentioned that the novelty is primarily coming from the chemistry being used in stripping, this aspect is not clear in the introduction and the claim seems misleading. Please clearly mention in the introduction how the authors' method is superior to other multiplexed imaging in terms of time, signal to noise ratio and cost of imaging?

We have updated the Introduction to better clarify the approach, and to highlight how HIFI differs from existing cyclic-IF approaches. Importantly, HIFI does not 'strip' antibodies, which requires harsh detergents. Instead, HIFI uses 2 synergistic strategies to remove markers: first, it employs modified low-power imaging to prevent oxidative cross-linking of fluorophores to the tissue, and second, it denatures antibodies with an extremely short wash to remove primary and secondary antibodies without disrupting the tissue. However, we do not claim that HIFI is superior in terms of imaging time, and cost of imaging to all other cyclic-IF methods. Other cyclic-IF approaches can be optimized to have similar imaging quality with off-the-shelf antibodies and reagents, especially when used on FFPE tissues. That is the benefit of cyclic-IF strategies. Our approach, along with the included pixel-by-pixel autofluorescence subtraction does improve signal-to-noise ratio, but what makes HIFI unique is that it maintains the benefits of cyclic-IF while eliminating the destructive nature inherent in the method.

There are also a range of critical improvements and optimizations to the cyclic-IF strategy that are included in the methodology, to which the quality of the data can attest. While it can be said that these improvements are not in and of themselves novel, they are no less valuable to the scientific community.

3. Authors mention: "Multiple days are required to complete a full hyperplexed panel. However, as most modern slide-scanning fluorescence microscopes have capacities for 80-100 slides, the ability to scale up to high-throughput offsets the total time required for an experiment when compared to other highly multiplexed approaches." However, comparing with other multiplexed IF approaches like CODEX, the timescale of staining and imaging still seem to be very slow as compared to existing MIF approaches.

PhenoCycler (CODEX) is certainly a powerful and well validated approach to high-dimensional imaging, especially if the experiment only requires a small number of slides. However, even with the new Fusion system, which can accommodate 2 slides together, it is only possible to image 10 samples a week with a 50-plex panel. With a standard fluorescent slide-scanner, such as the Zeiss Axio Scan.Z1 that we used in this study, 100 slides can be imaged in the same amount of time with the HIFI approach. So while HIFI is slower in terms of total time per single slide, it can scale to larger sample sets to become far more time efficient. This is why we do not claim HIFI is ‘superior’ to other approaches, only that is well suited to certain experimental scales that other approaches cannot achieve.

4. The Authors mention that the HIFI alignment tool is only limited by the memory (RAM) of the workstation. Since they are dealing with whole slide images, the RAM requirement would be very high. Please specify if the entire image is being loaded at once, if yes what are the specifications of the used workstation. This might significantly reduce the accessibility of this tool especially for large tissue sections.

We have updated the text to discuss the RAM specifications that were needed to achieve the images in the study, primarily to discuss that the ease of adoptability is dependent on the scale required (lines 236, 239). Upon public release, the Github page for the tool will include instructions for installing the tool on cloud servers, bypassing the need for onsite workstations.

5. Fig 3d- The authors selected areas of interest, however they initially discuss how selecting regions of interest can result in a selection bias and under sampling. Please clarify how their approach is different and unaffected by these limitations?

We have updated the manuscript to indicate that we do not introduce sampling bias in our image cropping because we include the entire lesion and affected areas of the surrounding brain in the region of interest. We believe we are not under-sampling the tumor microenvironment, which is the focus of the analysis, by cropping out large regions of healthy brain tissue. When we attempted spatial analysis of entire brain sections, the large number of healthy neuronal and glial cells heavily weighted downstream cluster analysis, unnecessarily complicating stratification of tumor microenvironment populations. We highlight the whole-slide capability of the technique for the benefit of those who would analyze much larger lesion areas, as we routinely do for patient sample imaging.

6. Fig 3e- “three biological replicates of rebound tumors demonstrated extensive intertumoral heterogeneity”. This is major biological insight authors draw from their results. Please explain this in more by detailing different features in the image and clearly marking those features in the image. What global insights can one draw from these differences that are useful for diagnostic purposes?

Based on this point, along with feedback from other reviewers, we have expanded our glioblastoma sample set to include n=5 biological replicates for each treatment condition. Representative images from each tumor are now shown in Supplemental Figure 3 to highlight the morphological heterogeneity. Unfortunately, because we harvest rebounded tumors when they became clearly detectable (as per our strict animal welfare guidelines) we cannot say how their morphological features correlate to survival or any other diagnostic endpoint. This is certainly a very interesting question which is unfortunately outside of the scope of this study.

7. Fig 4b- The authors should provide the accuracy achieved by their cell segmentation model trained using transfer learning

Based on this suggestion we have expanded the section relating to cell segmentation to include a larger example region (Figure 4b), comparisons to alternative approaches, and statistics for model accuracy. In

addition, we have collaborated with a consortium of imaging and machine learning experts to generate a new deep-learning cell segmentation model based on a large-scale initiative to create manually annotated ground-truth training data from multiple murine tumors and healthy organ tissues. Both the training data and segmentation model will be publicly released as a community resource if the manuscript is accepted for publication.

8. Authors should explain the biological insights gained from Leiden clustering. It seems like authors have described the methodology of Leiden clustering, but did not explain what they learned from it?

In addressing other reviewer requests for a larger sample set, we had to abandon our Leiden clustering approach for the revision as it struggled with the massive size of the expanded dataset. We instead optimized a more computationally efficient approach based on FlowSOM that greatly benefited from the dataset size in terms of increased accuracy and resolution (Supplemental Figure 4). This allowed us to identify more cellular phenotypes, which we could correlate with spatial patterning (Supplemental Figure 5 and 6). This is now discussed in the manuscript (lines 314-374).

9. Was there stage drift during multiple cycles of probe hybridization, imaging and washing in workflow? How did the authors detect and correct for it?

Dealing with stage drift was one of the initial challenges we needed to overcome when it came to image registration and alignment. This was achieved by developing a novel Python application of the pyramidal sub-pixel registration algorithm, with input from the original author, Philippe Thévenaz at EPFL. We have updated the section regarding the HIFI Alignment tool to better clarify this.

10. Was there any leaking of fluorescent light from one channel to another? What controls were done to ensure no spectral mixing?

All of these optimizations were performed prior to the study to ensure proper matches between fluorophores, and emission and excitation filters. We previously discussed in the manuscript that the low-power imaging used in HIFI further improves fluorescent signal specificity. The combination of these optimizations ensured no fluorescent signal leakage between channels.

11. Authors should show controls for following:
o Minimization of autofluorescence.
o Effectiveness of washing of probes.
o Minimal off target effects.

To address this suggestion, we have now included in the revised manuscript a control experiment that shows the elution efficiency of the approach (Supplemental Figure 1b). We also discuss in more detail how antibodies are validated for inclusion in the panel, and how the HIFI Alignment tool removes endogenous tissue autofluorescence.

Reviewer #3 - GBM models, imaging techniques - (Remarks to the Author):

This paper describes an innovative, practical, achievable-for-most-labs, multiplexed imaging approach that enables characterisation of the microenvironment in the spatial dimension. The innovation lies in its ease of use, its compatibility with routine laboratory equipment and reagents, and the development and implementation of software solutions that the authors have vetted in a unique preclinical mouse model of glioma. This set of tools will enable new discovery science in solid cancer biology, in both clinical and preclinical settings. Tools like this, with a low barrier to entry, are much needed to expand the scope of research in tumour-microenvironment interactions, specially the approach the authors have taken, which

analyses the TME in an experimentally manipulable model-- crucial to identify features that change with treatment or other interventions. Techniques like imaging mass cytometry and CODEX/Akoya require expensive equipment and reagents, or take a lot longer for data acquisition and analysis. Other spatial technologies like Visium have high depth of coverage, but perhaps that's overkill/not necessary for simply calling and phenotyping cell types accurately. This approach strikes the right balance and will be of great interest to many, perhaps not just to cancer biologists, but to developmental biologists and generally anybody interested in assaying the spatial context using a manageable 45-plex panel of commercially available antibodies.

We are very grateful to the reviewer for their positive assessment of the manuscript and approach. One of our primary motivations for this paper was to lower the barrier of entry for high dimensional imaging and spatial analysis, as these are incredibly useful tools for many fields of research. We are greatly encouraged that the reviewer shares this sentiment. The reviewer additionally provides several helpful suggestions, which we have now integrated into the study. We believe addressing these suggestions has further improved the manuscript.

We will address each of their comments below.

Having said that, can the authors comment on whether:

1) They could test this system on one additional model, perhaps not to the same level of detail as they have done the PDGfp model. Either another GEMM harbouring different mutations, or perhaps a metastatic brain tumour model. To evaluate whether differences between tumour genotypes, or between primary vs metastatic tumours, can be resolved using this system.

We found this to be an excellent suggestion, as the comparison of how different tumor types in the same tissue respond to treatment is indeed of great interest. We chose to perform a matched experimental design in a syngeneic model of breast-to-brain metastasis (BrM) utilizing the PyMT-derived 99LN model that we have used in previous publications (e.g. Croci et al, 2022). These tumor cells were implanted in the brain so as to have the same spatial coordinates, similar time of progression, and matched size to our genetic glioblastoma model. Tumors were treated with focalized radiotherapy, and harvested at 7 days post-treatment, or at the time of detectable regrowth (by MRI).

We observed substantial differences in the spatial reorganizational response to radiotherapy between glioblastoma and metastasis models, despite receiving an equivalent survival benefit from treatment (Figure A below, for reviewers only). We believe that these comparisons will be of greater potential interest to readers than the initial focus on GBM only in the original submission, and so have restructured the manuscript and figures to highlight these important differences.

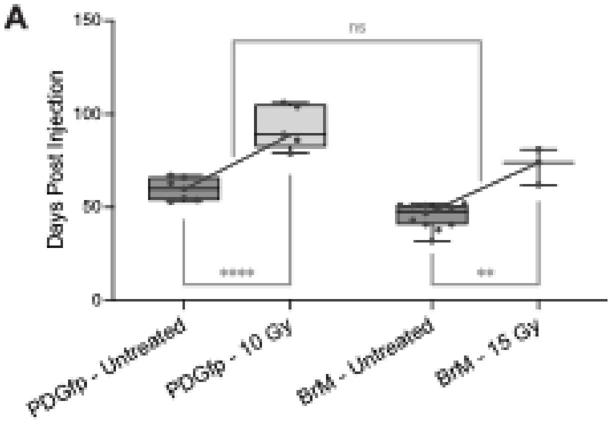

Figure A. Progression-free-survival of rebound PDGfp (n=5) and BrM (n=3) mice used in the study compared to historical data of untreated mice for each model. n=7 PDG mice from Akkari et al., Science Trans Med 2020, n=12 BrM from Wischnewski, Joyce et al., manuscript in preparation.

2) They could test the compatibility of this system for evaluating genotype:phenotype clonal relationships in highly intratumourally heterogeneous cancers, using a barcoding strategy delivered with the virus in vivo.

This is also a very interesting suggestion, but it is unfortunately out of the scope of the current study.

3) Whether experimental therapies that impact the TME (CSF1Ris) or the immune axis (CAR-Ts), change cellular neighbourhood associations.

We completely agree that these would be extremely interesting and important experiments. Indeed, this is part of an independent study that is also currently under revision.

4) Functional implications of the very interesting switch from CN13 (MDM) to CN6 (MG) in recurrent tumours.

In addressing another reviewer's suggestion to expand the sample size of the glioblastoma dataset, we added additional biological replicates (n=5 for each) that necessitated an improved method of cell classification to handle so many single cell objects (Supplemental Figure 4, 5). This approach increased the granularity of our analyses, and allowed us to identify additional phenotypic subtypes of macrophages and microglia in the microenvironment. The cell neighborhoods 13 and 6 in the initial clustering revealed substantial alterations in the perivascular niche of rebounded tumors, which we can now observe with even greater clarity in the current analysis (Figure 6a, 6c, Supplemental Figure 7). However, addressing the functional implications of this phenotypic switch would be challenging within the scope and timescale of this project.

By switching the focus of the analysis to comparisons of how glioblastoma and metastasis models respond to radiotherapy, we were able to highlight an even more functionally relevant observation in glioma samples 7 days post-treatment. In brief, we observed residual surviving dormant tumor cells residing in a distinct spatial niche that consistently formed after treatment. This niche had significant associations between surviving non-proliferative tumor cells, fibroblasts, reactive astrocytes, and infiltrative immune cells in a fibrotic local environment that correlates with previously observed features of dormancy-promoting niches (Figure 6). We believe the functional implications of dormancy-promoting environments to be much clearer than the previously highlighted alterations in the perivascular niche of rebound tumors.

These suggestions could well be beyond the scope of this manuscript, but some small attempt to provide a functional or mechanistic insight using this technique should be made, as it would greatly add to the impact this paper can have.

We agree with this suggestion, and believe that the addition of the brain metastasis model along with the comparison of TME response to radiotherapy in these tumors versus glioblastomas provides a series of new biological insights in the revised manuscript. We hope that this will help address the reviewer’s point.

Reviewers' Comments:

Reviewer #1:
Remarks to the Author:
In this much-improved ms, the authors have addressed all major limitations of the study.

Reviewer #2:
Remarks to the Author:
All reviewer comments have been addressed. The manuscript can be accepted for publication.

Reviewer #3:
Remarks to the Author:
I'm satisfied with the revisions that have been made.
